# Development and Promotion of an mHealth App for Adolescents Based on the European Code Against Cancer: Retrospective Cohort Study

**DOI:** 10.2196/48040

**Published:** 2023-11-28

**Authors:** Meritxell Mallafré-Larrosa, Ginevra Papi, Antoni Trilla, David Ritchie

**Affiliations:** 1 Association of European Cancer Leagues Brussels Belgium; 2 Faculty of Medicine and Health Sciences University of Barcelona Barcelona Spain

**Keywords:** adolescent health, cancer prevention, digital health, ECAC, European Code Against Cancer, health promotion, mHealth, mobile app, mobile health, NCD, noncommunicable disease, primary prevention

## Abstract

**Background:**

Mobile health technologies, underpinned by scientific evidence and ethical standards, exhibit considerable promise and potential in actively engaging consumers and patients while also assisting health care providers in delivering cancer prevention and care services. The WASABY mobile app was conceived as an innovative, evidence-based mobile health tool aimed at disseminating age-appropriate messages from the European Code Against Cancer (ECAC) to adolescents across Europe.

**Objective:**

This study aims to assess the outcomes of the design, development, and promotion of the WASABY app through a 3-pronged evaluation framework that encompasses data on social media promotion, app store traffic, and user engagement.

**Methods:**

The WASABY app’s content, cocreated with cancer-focused civil society organizations across 6 European countries, drew upon scientific evidence from the ECAC. The app’s 10 modules were designed using the health belief model and a gamification conceptual framework characterized by spaced repetition learning techniques, refined through 2 rounds of testing. To evaluate the effectiveness of the app, we conducted a retrospective cohort study using the WASABY app’s user database registered from February 4 to June 30, 2021, using a 3-pronged assessment framework: social media promotion, app store traffic, and user engagement. Descriptive statistics and association analyses explored the relationship between sociodemographic variables and user performance analytics.

**Results:**

After extensive promotion on various social media platforms and subsequent traffic to the Apple App and Google Play stores, a sample of 748 users aged between 14 and 19 years was included in the study cohort. The selected sample exhibited a mean age of 16.08 (SD 1.28) years and was characterized by a predominant representation of female users (499/748, 66.7%). Most app users identified themselves as nonsmokers (689/748, 92.1%), reported either no or infrequent alcohol consumption (432/748, 57.8% and 250/748, 33.4%, respectively), and indicated being physically active for 1 to 5 hours per week (505/748, 67.5%). In aggregate, the app’s content garnered substantial interest, as evidenced by 40.8% (305/748) of users visiting each of the 10 individual modules. Notably, sex and smoking habits emerged as predictors of app completion rates; specifically, male and smoking users demonstrated a decreased likelihood of successfully completing the app’s content (odds ratio 0.878, 95% CI 0.809-0.954 and odds ratio 0.835, 95% CI 0.735-0.949, respectively).

**Conclusions:**

The development and promotion of the WASABY app presents a valuable case study, illustrating the effective dissemination of evidence-based recommendations on cancer prevention within the ECAC through an innovative mobile app aimed at European adolescents. The data derived from this study provide insightful findings for the implementation of Europe’s Beating Cancer Plan, particularly the creation of the EU Mobile App for Cancer Prevention.

## Introduction

### Background

Cancer cases are on the rise due to changes in demographics and exposure to risk factors, adding to the significant financial costs already linked to the disease [1]. Europe has a tenth of the world’s population but accounts for a quarter of the world’s cancer cases. In 2020, a total of 2.7 million people in the European Union (EU) were diagnosed with the disease, and another 1.3 million people lost their lives to it [2]. Moreover, in 2018, the financial burden of cancer in Europe due to health expenditure, loss of productivity, and informal care costs was €199 billion (US $213 billion) [3]. Unless we take decisive action, the number of lives lost to cancer in the EU is set to increase by more than 24% by 2035, making it the leading cause of death in the EU [4]. The significant expected increase in the number of cancers demands measures to encourage the prevention of the disease.

The European Code Against Cancer (ECAC) [5] has been a key health literacy measure used by the public and third sectors since the 1980s to promote and mainstream cancer prevention [6]. The ECAC, which is a trusted preventive tool free of commercial influence providing a reliable synthesis of the latest scientific evidence on cancer prevention, suggests that around 40% of cancers in Europe could be prevented through a mix of individual- and population-level actions known to be effective [5]. The current fourth edition of the ECAC aims to inform people about how to avoid or reduce their exposure to carcinogens, adopt behaviors that can lower their risk of developing cancer, and participate in organized screening programs through 12 easy-to-follow recommendations that do not require any special skills or advice [5]. The available evidence that cancer can be greatly prevented in Europe, coupled with support from the World Health Organization (WHO) for an inclusive, life-course approach to cancer prevention in its worldwide action plan for the prevention and control of noncommunicable diseases (NCDs) [7], sets a strong case for targeting adolescents and young people to multiply the benefits [8].

### The Importance of Adolescent Health for Cancer Prevention

Adolescence, as defined by the WHO, spans from the 10th to the 19th year of life and represents a period characterized by rapid and pivotal growth and transformation, second only to infancy [9]. During this life stage, individuals undergo substantial changes in their physical, cognitive, and psychosocial development. This is a crucial phase for the establishment of positive habits and the development of behaviors that can exert a lasting influence on both their current and future health, as well as the health of their potential children [10].

The welfare of adolescents varies considerably across European countries [11]. Some of the health issues they face are associated with their lifestyles and risky behaviors, including alcohol and tobacco consumption, as well as sedentary and poor dietary habits [12,13]. Consequently, enhancing adolescents’ awareness of the prevention messages within the ECAC and how modifiable lifestyle factors can influence cancer risk is imperative for shaping their lifelong patterns of healthy behavior.

To grow and develop in good health, adolescents require access to information, including age-appropriate comprehensive cancer prevention education. It is widely recognized that adolescents heavily rely on web-based information; however, they frequently fall victim to misinformation concerning modifiable risk factors and healthy lifestyles [14]. Moreover, their strategies for evaluating information tend to be unsophisticated and inadequate [15]. This underscores the importance of offering them easily accessible, robust, and evidence-based information.

### Mobile Health Technologies for Cancer Prevention

Mobile health (mHealth) technologies, underpinned by scientific evidence and ethical standards, exhibit considerable promise and potential in actively engaging consumers and patients while also assisting healthcare providers in delivering evidence-based care across the cancer control continuum [16]. This is substantiated by the WHO, which acknowledges that digital tools are an asset in supporting healthy lifestyles and addressing NCDs [17].

Numerous mobile apps with a focus on cancer often emphasize patient empowerment and self-care [18,19] or concentrate on addressing specific risk factors and types of cancer [20,21]. Hence, these apps may not inherently suit the context of healthy adolescents. Regarding concerns on the effectiveness of app-based interventions in promoting healthier lifestyles, the results are mixed and heavily reliant on the primary recommendations being conveyed. For young adults, these interventions have proven to be successful in promoting smoking cessation [22], improving dietary habits [23], managing weight [24], and reducing alcohol consumption [25]. Adolescents have also benefited from digital tools, particularly in terms of improving their diet [26-28] and promoting sun protection habits [29,30]. Additionally, positive results have been observed when using apps that target multiple health risks simultaneously, both in review studies [31,32] and primary research [33,34]. However, these apps lack comprehensiveness in addressing the entirety of modifiable risk factors recognized by the ECAC.

Considering the widespread adoption of mobile technology among adolescents and the findings from the literature mentioned above, leveraging smartphone technology to promote behaviors that enhance adolescents’ health literacy regarding cancer risk factors appears promising. Therefore, we developed a novel mobile app (WASABY) to encourage the adoption of a healthy lifestyle for the purpose of cancer prevention within the adolescent subpopulation.

### App Rationale

The WASABY app (hereafter “app”) was developed by the Association of European Cancer Leagues (ECL) as an evidence-based, educational mHealth tool to facilitate the dissemination and comprehension of age-appropriate messages outlined in the ECAC to a demographic of healthy adolescents within Europe, spanning the age range of 14-19 years. In particular, the app was designed to impart knowledge on modifiable cancer risk factors and guidance on mitigating individual risk in a fun and interactive way. Importantly, it does not dispense medical advice for patients with cancer or any other vulnerable or ill populations.

The app was primarily devised with the intention of being seamlessly integrated into preexisting or new health promotion and cancer education programs and interventions carried out by cancer-focused civil society organizations (hereafter “cancer leagues”) across Europe. Indeed, despite being publicly available for download in the Google Play and Apple App stores, the ECL did not intend solely to develop a new app; rather, we wished to enhance the effectiveness and reach of cancer leagues’ initiatives and provide them with a valuable tool for assessing knowledge acquisition regarding the ECAC at no cost.

In a subsequent phase, the ultimate goal would be to determine whether the integration of the WASABY app into cancer leagues’ multidimensional interventions can effectively foster the adoption of evidence-based cancer prevention recommendations among the adolescent demographic.

### App Description

Within the WASABY app, users are guided through the completion of 10 interactive modules designed to dispel common cancer prevention myths. Each module is structured around one of the prevention recommendations from the ECAC and features a combination of videos, practical tips, and interactive quizzes.

**Figure 1:**
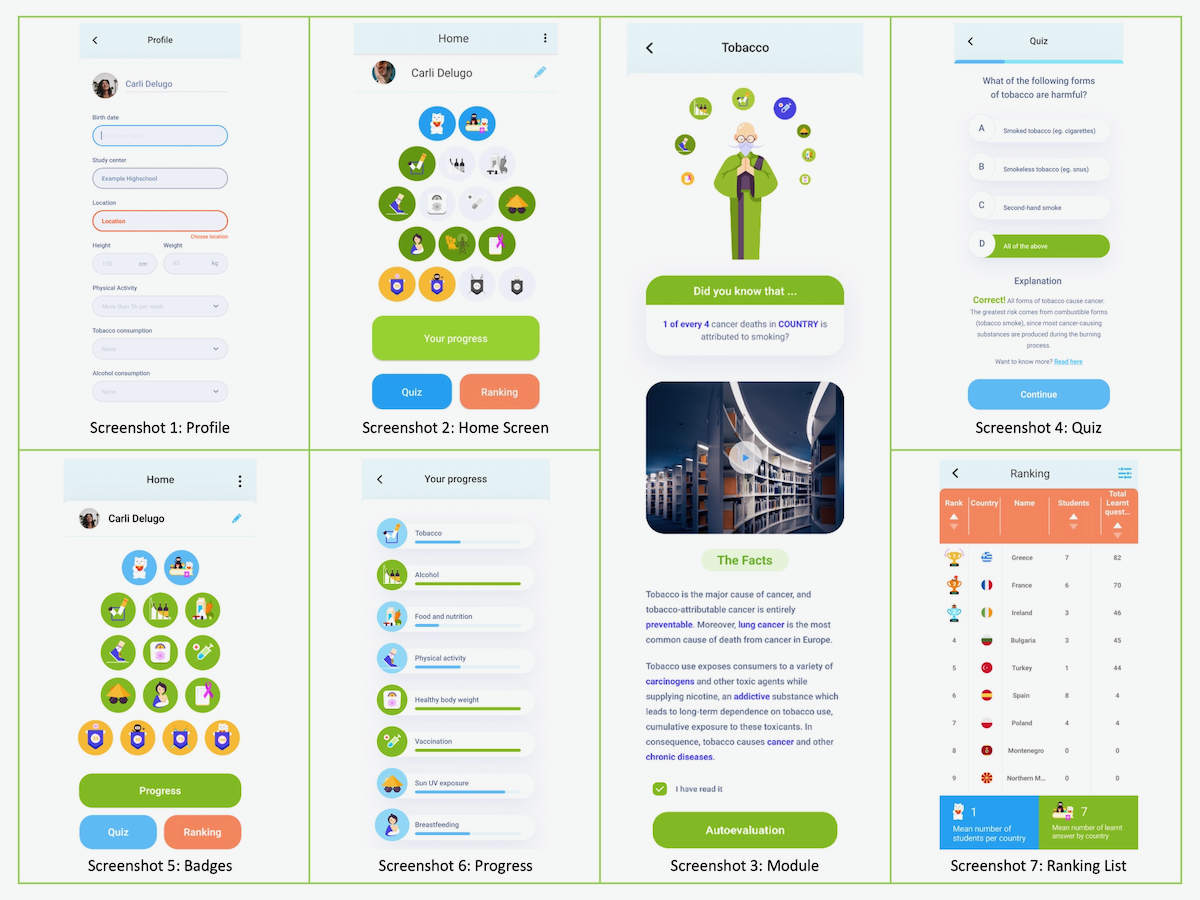
Structure overview of the WASABY app.

In compliance with EU privacy regulations, users are required to create a personal account and insert their personal details and lifestyle factors in order to access the app (Figure 1, screenshot 1). Once logged in, users can navigate the app from the home screen, as shown in screenshot 2 in Figure 1. From the home screen, users can access any of the 10 interactive modules, where they can read practical recommendations, view engaging videos, and participate in interactive quizzes (Figure 1, screenshot 3). Each module consists of 4 sections: a teaser question, a short introductory video, easily digestible facts, and a self-assessment quiz. The self-assessment quizzes are made up of 3 questions (Figure 1, screenshot 4). Users receive detailed explanations upon selecting their responses.

Returning to the home screen, users can review their profiles, earn reward badges, and track their progress. Additionally, they can access the final quiz and the ranking of the top learners. Regarding the badges, the app uses an incentive-based mechanism wherein achievement badges are unlocked upon completing each module, with icons becoming colorful as users advance in their learning (Figure 1, screenshot 5). By clicking on the progress button, users can monitor which modules they have completed, have not started, or are currently ongoing (Figure 1, screenshot 6). Upon the completion of all modules, users have the opportunity to take a final quiz to test their knowledge.

Importantly, real-time progress data are recorded, enabling users to share their progress and quiz results with friends and other players. This contributes to the creation of an international ranking list of “top learners” across Europe (Figure 1, screenshot 7). These features foster a competitive spirit, instill a sense of accomplishment, and encourage the repeated use of the app.

### Aim of the Study

In this paper, we present the preliminary findings of the design, iterative development, and promotion of the WASABY app, made available to European adolescents in real-life settings. The objectives of this study were threefold: (1) to analyze data pertaining to the app’s promotion on social media, (2) to assess the traffic generated on Google Play and Apple App stores, and (3) to evaluate the level of user participation and engagement with the app.

## Methods

### WASABY App Development

The WASABY app’s content was developed by drawing upon the ECAC’s scientific evidence [5]. Specifically, a total of 6 cancer leagues located in Spain (Asociación Española Contra el Cáncer), the United Kingdom (Cancer Focus Northern Ireland), Slovenia (Zveza slovenskih društev za boj proti raku; Association of Slovenian Cancer Societies), France (La Ligue contre le Cancer), Switzerland (Krebsliga), and Romania (Societatea Româna de Cancer) were engaged in this process. Additionally, a total of 111 adolescents aged between 14 and 19 years from 25 EU Member States actively participated in 2 testing rounds.

First, a selection of relevant ECAC messages (specifically, ECAC messages 1-7 and 10-11) was made, taking into consideration the age group of the target audience. Second, materials sourced from the ECAC’s scientific website [35], which served as the foundation for the content of the app, were operationalized by applying the health belief model (HBM) [36]. The operationalization of the HBM within the WASABY app involved the strategic design and presentation of content that aligned with the core constructs of the model. The process involved integrating educational modules and interactive elements to raise awareness of the risks of unhealthy behaviors, emphasizing the benefits of adopting healthy habits, providing practical strategies to overcome barriers, and incorporating cues to action to boost users’ confidence in making positive lifestyle changes. Thus, the app’s content was crafted to influence users’ perceptions, attitudes, and intentions related to health behaviors. Consistent with similar apps assessed in the existing literature and using a methodology evocative of the well-known Duolingo Language app [37], the app was also grounded in a conceptual framework of gamification, characterized by spaced repetition learning techniques aimed at promoting efficient and effective learning, especially in achieving long-term information retention compared to concentrated massed practice [38].

Third, the 10 content modules and associated quizzes that resulted from the selection of specific ECAC messages and their operationalization through the HBM underwent a series of revisions, which were conducted by cancer leagues to assess their adequacy, comprehensibility, and accuracy. We used a structured approach to ensure the adequacy and accuracy of the content. Initially, we used the nominal group technique to collaboratively define the scope of each module. Subsequently, an iterative expert review process engaged specialists from both medical and educational domains within the ECL’s network of cancer leagues. These experts critically assessed and refined the content to enhance its clarity and alignment with user needs. Subsequent refinements were made to ensure that the messaging would be suitable for a reading level appropriate for 12-year-old children. This was achieved following beta and alpha tests with the app’s target population.

### WASABY App Testing

The beta version of the app was developed for Android devices and made available in the 27 EU member states (plus the United Kingdom). It underwent a first round of testing through a web-supported 19-item questionnaire to assess comprehension and suitability of the app’s content. From May 27 to June 10, 2020, a social media campaign was used to recruit 83 testers within the app’s target group (ie, healthy 14- to 19-year-olds residing in Europe) from 25 EU countries to participate in the beta test (Multimedia Appendix 1).

Originally developed in English, the app was later translated into 6 additional languages (French, German, Italian, Romanian, Slovenian, and Spanish) and adapted to be used on iOS devices. It underwent a second round of testing (alpha test) to check for functionality and technical aspects through a dedicated 18-item questionnaire, enrolling a total of 28 testers: 4 individuals per language and 2 per platform (Android and iOS). The final version of the app included feedback implemented from the 2 subsequent rounds of testing and was made available in all countries of the WHO Europe region in both the Google Play and Apple App stores.

### Study Design and Population

We conducted a retrospective cohort study using data from the app’s database of registered users, covering the period from February 4 to June 30, 2021. The sample size was determined based on the available retrospective cohort of 976 registered users. Exclusion criteria were applied to users outside of the age target group (14- to 19-year-olds) and those with invalid or partially missing registration data. Anonymized data were used for all analyses. This study adhered to the STROBE (Strengthening the Reporting of Observational Studies in Epidemiology) reporting guideline [39].

### WASABY App Evaluation Framework

A 3-pronged evaluation framework was developed with indicators reflecting the promotion of the app on social media, the traffic generated on Google Play and Apple App stores, and the level of engagement exhibited by app users. Instagram-powered analytics were monitored and analyzed monthly during the study period. Variables collected included: accounts reached, content interactions, profile visits, website taps, top posts, and follower-specific analysis. The app’s traffic in app stores was analyzed through Google- and Apple-powered key performance indicators (KPIs), including product page views, product installations, conversion rate, deletions, crashes, and average rating [40,41]. Such KPIs were stratified by country, date, and download source.

Variables from the user registry database were collected, including anonymized user identification, demographics (birth date, sex, country, region, and language), anthropometrics (height and weight, through which BMI was calculated), and self-reported cancer risk factors (physical activity, tobacco use, and alcohol consumption). The database also contained information on the completion of the app’s modules according to 3 variables (visits, readings, and completed auto-evaluation). Variables were operationalized to serve as proxies for the following constructs: content interest, content completion, and quiz completion (Table 1 presents details on variable definition and assessment). Overall, the app as a tool was considered completed upon 100% module reading registry.

**Table 1:** Variables capturing WASABY mobile app individual user performance. Each variable was assessed separately for each of the app’s 10 content modules.

Variable	Construct	Definition and interpretation
Module visits	Content interest	Variable registering the amount of page visits into a specific module. Interest was operationalized as a continuous variable, by which greater values capture greater interest.
Module readings	Content completion	Variable registering the click on “I have read it” button present at the end of each module. Content completion was operationalized as a dichotomous variable, considered complete if 1 or more readings were recorded.
Completed autoevaluations	Quiz completion	Variable registering the number of completed quiz questions per module (7 available per module, with unlimited response opportunities). Quiz completion was operationalized as a dichotomous variable, by which a given module’s autoevaluation was considered complete if 3 or more questions were registered.

### Statistical Analysis

We performed descriptive statistics based on frequencies (for categorical variables) and mean and median values (including SD for continuous ones). Statistical differences among users’ app completion (outcome variables) according to demographic (age and sex) and self-reported risk factors (tobacco, alcohol, and physical activity; independent variables) characteristics were tested using the Mann-Whitney Wilcoxon test at .05 significance level. Outcome variables were treated as continuous (details on their operationalization are in Table 1). Odds ratio (OR) and 95% CI were used to assess the interrelation of the independent variables mentioned above with a proxy for the app’s completion. The WASABY app was considered completed upon 100% of the module reading, and thus the outcome variable was dichotomized. All statistical analyses were performed using the R software (version 4.2.1; R Foundation for Statistical Computing).

### Ethical Considerations

This project received ethical approval through the WASABY project consortium (EC PP-2-5-2016). Data collection and storage were managed by Adhere Health Inc (formerly Salumedia Tecnologías S.L.U). The storage of the database adhered to the General Data Protection Regulation (GDPR) and the corresponding Spanish regulation. Correspondingly, a privacy policy, legal notice, and terms of use were formulated (Multimedia Appendix 2). All participants agreed to the terms of use upon app registration independently, that is, without parental approval being required. All data from the WASABY app registration database were obtained in anonymized form for the purposes of the analysis hereby presented.

## Results

The results we present below have been organized according to the 3 components of the app’s evaluation framework.

### Social Media Promotion

A 10-day social media campaign, beginning on World Cancer Day (February 4, 2021), was run on Instagram to launch and promote the app. The boosted social media posts reached 851,149 people and received 2,470,418 impressions. Subsequently, the app was promoted again during European Week Against Cancer (May 25-31, 2021) through an organic social media campaign, which received 3799 impressions, as well as GDPR-compliant targeted emails sent to over 100 contacts within the ECL’s network of cancer leagues and youths. As of June 30, 2021, the app’s dedicated web page on the ECL’s website [42] had been visited 10,315 times.

### WASABY App Store Traffic

Between January and June 2021, the app received a total of 3426 impressions on both the iOS and Android stores, resulting in 1109 downloads. This translates to a 32.37% (1109/3426) conversion rate, which was largely influenced by the World Cancer Day and European Week Against Cancer promotional web-based campaigns. Over the same period, 645 app deletions were reported, which are to be contextualized given the 2-week completion time frame under which the app was designed. Additionally, on the iOS platform, an average of 3.32 sessions per active user were recorded. A summary of the app stores’ KPIs is found in Multimedia Appendix 3.

### WASABY App User Engagement

During the study period, a total of 976 users were fully registered in the app’s database. After applying all inclusion and exclusion criteria, 748 users aged between 14 and 19 years were included in the study cohort. Table 2 includes a summary of the sample demographics. As more than half (n=392, 52.4%) of the sample was composed of users from Slovenia, this subgroup is reported separately.

Overall, the mean age was 16.08 (SD 1.28) years with a median of 16 years, similar to Slovenia’s cohort (mean 16.31, SD 3.83; median 16 years). Female users were overrepresented, accounting for 66.7% (499/748) of all users (Slovenia: 281/392, 71.7%). The app’s interface was predominantly accessed in English (351/748, 46.9%) or Slovenian (350/748, 46.7%). Self-reported anthropometric data were used to estimate BMI, and the cohort had a mean of 21.86 (SD 4.18) kg/m^2^. Approximately 72.1% (539/748) of users fell within the 18-25 kg/m^2^ range, which is considered normal according to international standards.

Self-reported behavioral risk factors related to tobacco smoking, alcohol consumption, and physical activity were collected upon registration (Table 3). Most users identified themselves as nonsmokers (689/748, 92.1%) and reported either no or infrequent alcohol consumption (432/748, 57.8% and 250/748, 33.4%, respectively). Moreover, 67.5% (505/748) of users indicated being physically active for 1-5 hours per week.

**Table 2:** Demographics of users registered in the WASABY app database.

Demographics			Overall (N=748), n (%)		Slovenia (n=392), n (%)
**Age** **(years)**					
	14	76 (10.2)		8 (2)	
	15	200 (26.7)		103 (26.3)	
	16	186 (24.9)		104 (26.5)	
	17	187 (25)		123 (31.4)	
	18	71 (9.5)		43 (11)	
	19	28 (3.7)		11 (2.8)	
**Sex**					
	Female	499 (66.7)		281 (71.7)	
	Male	179 (23.9)		63 (16.1)	
	Unreported	70 (9.4)		48 (12.2)	
**Country^a^**					
	Belgium	7 (0.9)		N/A^b^	
	Bulgaria	16 (2.1)		N/A	
	Czechia	13 (1.7)		N/A	
	Denmark	1 (0.1)		N/A	
	Germany	13 (1.7)		N/A	
	Estonia	12 (1.6)		N/A	
	Ireland	12 (1.6)		N/A	
	Greece	14 (1.9)		N/A	
	Spain	14 (1.9)		N/A	
	France	5 (0.7)		N/A	
	Croatia	23 (3.1)		N/A	
	Italy	25 (3.3)		N/A	
	Latvia	21 (2.8)		N/A	
	Lithuania	17 (2.3)		N/A	
	Luxembourg	3 (0.4)		N/A	
	Hungary	12 (1.6)		N/A	
	Malta	5 (0.7)		N/A	
	The Netherlands	9 (1.2)		N/A	
	Austria	2 (0.3)		N/A	
	Poland	34 (4.6)		N/A	
	Portugal	13 (1.7)		N/A	
	Romania	49 (6.6)		N/A	
	Slovenia	392 (52.4)		N/A	
	Slovakia	13 (1.7)		N/A	
	Finland	5 (0.7)		N/A	
	Sweden	6 (0.8)		N/A	
	United Kingdom	10 (1.3)		N/A	
	Switzerland	1 (0.1)		N/A	
	Northern Macedonia	1 (0.1)		N/A	
**Language interface**					
	English	351 (46.9)		46 (11.7)	
	Spanish	11 (1.5)		0 (0)	
	Italian	18 (2.4)		0 (0)	
	German	16 (2.1)		0 (0)	
	Slovenian	350 (46.8)		346 (88.3)	
	Romanian	2 (0.3)		0 (0)	
	French	6 (0.8)		0 (0)	
**Height** **(cm)**					
	130-140	1 (0.1)		1 (0.3)	
	140-150	2 (0.3)		1 (0.3)	
	150-160	61 (8.2)		28 (7.1)	
	160-170	330 (44.1)		182 (46.4)	
	170-180	257 (34.4)		134 (34.2)	
	180-190	82 (11)		40 (10.2)	
	190-200	11 (1.5)		5 (1.3)	
	Unreported	4 (0.5)		1 (0.3)	
**Weight** **(kg)**					
	30-40	2 (0.3)		0 (0)	
	40-50	65 (8.7)		30 (7.7)	
	50-60	266 (35.6)		148 (37.8)	
	60-70	231 (30.9)		115 (29.3)	
	70-80	102 (13.6)		56 (14.3)	
	80-90	48 (6.4)		26 (6.6)	
	90-100	18 (2.4)		9 (2.3)	
	100-110	8 (1.1)		4 (1)	
	110-120	2 (0.3)		2 (0.5)	
	120-130	6 (0.8)		2 (0.5)	
**BMI (kg/m^2^)**					
	10-18	89 (11.9)		35 (8.9)	
	18-20	180 (24.1)		94 (24)	
	20-25	359 (48)		203 (51.8)	
	25-30	84 (11.2)		43 (11)	
	30-35	18 (2.4)		9 (2.3)	
	35-40	7 (0.9)		4 (1)	
	>40	7 (0.9)		3 (0.8)	
	Unknown	4 (0.5)		1 (0.3)	

^a^8 countries within the World Health Organization Europe region were excluded, given there were no registered users in the WASABY app database (Cyprus, Iceland, Liechtenstein, Norway, Montenegro, Albania, Serbia, and Turkey).

^b^N/A: not applicable.

**Table 3:** Self-reported risk factors upon use registration in the WASABY app.

Self-reported risk factors			Overall (N=748), n (%)		Slovenia (n=392), n (%)
**Tobacco use** **(cigarettes per day)**					
	None	689 (92.1)		361 (92.1)	
	1-5	38 (5.1)		22 (5.6)	
	5-10	14 (1.9)		8 (2)	
	10-20	4 (0.5)		0 (0)	
	≥20	3 (0.4)		1 (0.3)	
**Alcohol consumption** **(frequency)**					
	None	432 (57.8)		221 (56.4)	
	Rarely	250 (33.4)		131 (33.4)	
	Only on weekends	50 (6.7)		27 (6.9)	
	Often	12 (1.6)		10 (2.6)	
	Everyday	4 (0.5)		3 (0.8)	
**Physical activity** **(approximate hours per week)**					
	Sedentary	70 (9.4)		28 (7.1)	
	1	125 (16.7)		61 (15.6)	
	3	238 (31.8)		121 (30.9)	
	5	142 (19)		84 (21.4)	
	>5	173 (23.1)		98 (25)	

The individual and overall app’s performance was investigated through 3 variables (defined in Table 1 and results presented in Table 4). In aggregate, the app’s content garnered substantial interest, as evidenced by 40.8% (305/748) of users accessing each of the 10 individual modules. Similarly, a comparable proportion of users completed the modules, with 36.9% (276/748) reading all of them and 34.5% (258/748) finishing all self-assessment quizzes. Notably, Slovenian users demonstrated the highest level of engagement: they were most likely to access all modules (190/392, 48.5%), read the modules’ contents (167/392, 42.6%), and complete the quizzes (145/392, 37%).

**Table 4:** WASABY app performance metrics in terms of app interest, content completion, and quiz completion (operationalized variable description available in Table 1).

App use constructs			Overall (N=748), n (%)		Slovenia (n=392), n (%)
**App interest** **(number of modules visited)**					
	0	36 (4.8)		31 (7.9)	
	1	101 (13.5)		43 (11)	
	2	75 (10)		30 (7.7)	
	3	72 (9.6)		22 (5.6)	
	4	47 (6.3)		18 (4.6)	
	5	48 (6.4)		23 (5.9)	
	6	33 (4.4)		18 (4.6)	
	7	14 (1.9)		6 (1.5)	
	8	7 (0.9)		2 (0.5)	
	9	10 (1.3)		9 (2.3)	
	10 (all)	305 (40.8)		190 (48.5)	
**Content completion** **(number of modules read)**					
	0	139 (18.6)		75 (19.1)	
	1	86 (11.5)		30 (7.7)	
	2	62 (8.3)		27 (6.9)	
	3	53 (7.1)		19 (4.9)	
	4	41 (5.5)		17 (4.3)	
	5	35 (4.7)		18 (4.6)	
	6	27 (3.6)		18 (4.6)	
	7	11 (1.5)		7 (1.8)	
	8	6 (0.8)		2 (0.5)	
	9	12 (1.6)		12 (3.1)	
	10 (all)	276 (36.9)		167 (42.6)	
**Quiz completion** **(number of modules with quiz completed)**					
	0	171 (22.9)		88 (22.5)	
	1	83 (11.1)		29 (7.4)	
	2	61 (8.2)		31 (7.9)	
	3	62 (8.3)		31 (7.9)	
	4	37 (5)		16 (4.1)	
	5	29 (3.9)		17 (4.3)	
	6	18 (2.4)		10 (2.6)	
	7	9 (1.2)		5 (1.3)	
	8	6 (0.8)		6 (1.5)	
	9	14 (1.9)		14 (3.6)	
	10 (all)	258 (34.5)		145 (37)	

Significant differences were observed by sex in terms of the number of modules visited, read, and quizzes completed (*P*=.02, *P*=.047, and *P*=.03, respectively), with male users being less likely to complete the overall app (OR 0.878, 95% CI 0.809-0.954). Conversely, there were no differences found by age group (dichotomized as 14-16 years vs 17-19 years) in the abovementioned tested associations.

**Figure 2:**
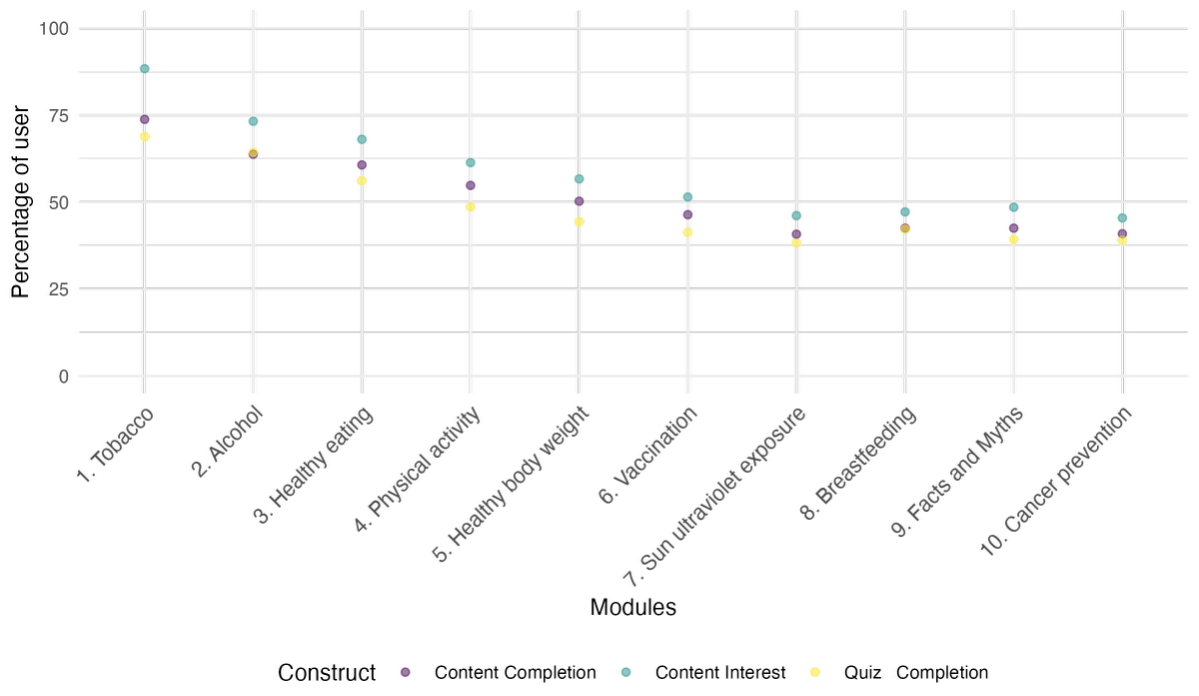
WASABY app performance metrics by module (1-10) in terms of app interest, content completion, and quiz completion.

Additionally, significant variations were noted in the abovementioned associations concerning self-reported user risk factors based on dichotomized tobacco consumption (*P*=.04, *P*=.07, and *P*=.03). Self-reported tobacco users demonstrated a reduced likelihood of completing the app (OR 0.835, 95% CI 0.735-0.949). No notable distinctions were detected concerning alcohol consumption or physical activity. Finally, while evaluating the app’s performance based on individual modules, a decreasing linear relationship was observed while progressing through module 1 (on tobacco) to module 10 (on cancer prevention; Figure 2).

## Discussion

### Principal Findings

The results of the WASABY app pilot study have demonstrated the potential of an mHealth app to promote evidence-based cancer prevention recommendations to European adolescents. While most mHealth apps addressing cancer prevention have focused on specific risk factors (such as body weight [43]) or specific cancer sites (such as breast cancer [44]) and a plethora of interventions targeting patients with cancer and survivors of cancer have been developed [45], there is currently no other comprehensive app based on the ECAC that specifically targets adolescents aged between 14 and 19 years, to the best of our knowledge.

The app was successful in engaging a large proportion of users across all its modules, with 40.8% (305/748) of users visiting all 10 modules. Similarly, 36.9% (276/748) of users completed each module, and 34.5% (258/748) completed the entire app autoevaluation assessment, indicating that over one-third of users in the pilot study completed the app. Given that the content of the app covers a wide range of cancer risk factors and protective measures as outlined in the ECAC, this encouraging result suggests that covering multiple domains of cancer prevention is feasible without deterring user interest and adherence.

As shown in Figure 2, a decreasing linear relationship was observed in the app’s completion across the 10 individual modules, with the highest level of interest and completion reported for module 1 (focused on tobacco), which gradually decreased until module 10 (focused on the ECAC). While it is reasonable to expect a decline in user retention across the modules as users progress through the app [46], the added value of the WASABY app concept lies in addressing the multiple recommendations of the ECAC. Therefore, if users discontinue using the app after completing the initial modules that focus on lifestyle-related risk factors, they will not benefit from the crucial knowledge related to cancer prevention, particularly myths and misconceptions (addressed in module 9), thereby reducing the potential impact of the app. The data from the pilot also showed that sex was a predictor of completion of the modules. This may be explained by an overrepresentation of female users, with approximately two-thirds of users identifying as female. Conversely, nonsmoker users were more likely to adhere throughout the content until the last module, underlining the importance of understanding the sociodemographics of the target audience to best target the messaging in novel digital health interventions [47]. Additionally, such characteristics shall be considered as well in the promotion and recruitment methods for app users to achieve a more representative reach among the target population.

Comparing the results of this study with the findings of previous studies in the literature becomes difficult when considering the small population sizes and heterogeneous designs of mHealth interventions. A systematic review and meta-analysis reported that eHealth school-based interventions addressing multiple lifestyle risk factors can be effective in improving physical activity and fruit and vegetable consumption, indicating the potential for multirisk factor application targeting adolescents [48]. An earlier scoping review on apps to promote healthy lifestyle among adolescents concluded that the ability to set personal goals enhances self-monitoring and increases awareness [49]. The review also determined that most apps were implemented as part of therapy or to strengthen school programs, supporting the original conceptual design of the pilot intervention for the WASABY app [49]. Additionally, a total of 2 umbrella reviews published in 2023 on digital interventions to moderate alcohol consumption in young people and physical inactivity and nutrition in young people [50] identified the potential of digital interventions to increase physical activity and improve nutrition in school-age children and reduce alcohol consumption in certain subpopulations of younger people, especially if active feedback is provided by the mHealth intervention. The overall body of evidence is characterized by substantial heterogeneity, inconsistent population groups, and intervention definitions. This indicates that the effectiveness of mHealth tools for health promotion may suffer from the small effects of interventions, which remain detectable for a short period of time after the conclusion of the intervention.

Finally, during the pilot period, the promotion strategy of the app relied partially on the support of nongovernmental organizations (NGOs) to increase awareness and ultimately integrate the app into their existing multidimensional health education programs. Cancer leagues are key NGOs acting as primary promoters of the ECAC at the national, regional, and local levels, marking them out as ideal promoters of the app. Cancer leagues were involved in the cocreation process from the early stages of the app’s development. Notably, the number of downloads was particularly influenced by the endorsement and promotion of the app through the national leagues, with users in Slovenia demonstrating the highest engagement rates across all modules. They were most likely to access all modules (190/392, 48.5%), read the modules’ contents (167/392, 42.6%), and complete the quizzes (145/392, 37%). This highlights the success of the Zveza slovenskih društev za boj proti raku (Association of Slovenian Cancer Societies) in adopting the WASABY app for youth-targeted initiatives and demonstrates that with committed support from a key stakeholder for the promotion of the app, it is possible to achieve good uptake.

### Limitations

There are several limitations that should be acknowledged. First, as this study was designed to evaluate the outcomes of the design, development, and dissemination of the WASABY app, the evaluation framework’s scope was limited in terms of time and reach. As a result, certain dimensions, such as knowledge acquisition and user retention, could not be adequately evaluated due to the lack of monitoring of KPIs over a longer period (ie, at 6 and 12 months after completion). Additionally, the fidelity of the tool implementation was impacted by the COVID-19 pandemic. The initial plan was to pilot the tool through in-person demonstration at existing health education outreach programs organized by cancer leagues in 6 European countries. However, the app’s promotion and dissemination had to be conducted entirely through social media channels. Therefore, much of the data collected for this study relied on self-reporting, and no measures were in place to validate user app registration. Additionally, due to the scope of the study analysis, which was rather exploratory, no adjustments by age or sex groups were conducted in the statistical analysis. Lastly, the data reported were insufficient to determine whether the app promotion was only reaching health-literate populations within the target group. It is, therefore, not possible to determine whether the app’s pilot reached a representative cross-section of the population or if it was installed and completed by individuals who were already more likely to comply with the recommendations of the ECAC. This would be a key area of further research in future studies on mHealth tools.

### Future Recommendations

The app was developed to promote and encourage adolescents to follow the ECAC recommendations. Evidence suggests that the ECAC is not well-known among the general public [51]. Therefore, the app could help to improve awareness and, subsequently, knowledge and adherence to these recommendations. With this objective in mind, the European Commission has mandated the development of the “EU Mobile App for Cancer Prevention” under Europe’s Beating Cancer Plan [52]. The results and lessons learned from the WASABY app should be taken into account for this new EU endeavor. To improve adherence and retention, future iterations of the app or comparable tools should further gamify its content, providing motivation and incentives to complete each module. It is also essential to consider the sociodemographic characteristics of the target population when promoting apps to ensure they reach a more diverse and representative population. Engaging with NGOs to cocreate and promote the WASABY app was beneficial, but further research is required to assess the feasibility of embedding the app as an intervention within a broader health education program. Furthermore, it is necessary to evaluate the impact of knowledge acquisition of the ECAC recommendations on the intention to adopt the recommendations in daily life.

### Conclusions

The experience gained from designing, developing, and promoting the WASABY app provides a valuable case study on the effective dissemination of evidence-based recommendations on cancer prevention within the ECAC through an innovative digital health tool aimed at European adolescents. The data obtained from this study show the potential of an mHealth app that addresses multiple risk factors, thus laying the groundwork for the creation of new tools to encourage healthy lifestyles and mitigate NCDs. The insights derived from the study also hold significance for the implementation of Europe’s Beating Cancer Plan, particularly the development of the “EU Mobile App for Cancer Prevention” [52].

## Appendix

Multimedia Appendix 1 WASABY App testing results from a web-supported 19-item questionnaire to assess comprehension and suitability of the App’s content.

Multimedia Appendix 2 Privacy policy, legal notice, and terms of use.

Multimedia Appendix 3 App stores' key performance indicators.

## Abbreviations

ECAC: European Code Against Cancer
ECL: Association of European Cancer Leagues
EU: European Union
GDPR: General Data Protection Regulation
HBM: health belief model
KPI: key performance indicator
mHealth: mobile health
NCD: noncommunicable disease
NGO: nongovernmental organization
OR: odds ratio
STROBE: Strengthening the Reporting of Observational Studies in Epidemiology
WHO: World Health Organization

## Data Availability

The WASABY app’s content is available on GitHub [53]. The data that support the findings of this study are available from the corresponding author upon reasonable request.
