# Supplementary material for: Development and Promotion of an mHealth App for Adolescents Based on the European Code Against Cancer: Retrospective Cohort Study
Source: JMIR Cancer. 2023 Nov 28;9:e48040. doi: 10.2196/48040 (PMC10716759; doi:10.2196/48040)
Supplement: Multimedia Appendix 1 [file cancer_v9i1e48040_app1.pdf]

WASABY

BETA TESTING | Feedback analysis

BETA TESTERS | Population demographics (I)

Indicator	Count
Interested beta testers	260
Final beta testers	83
Eligible for the EWAC prize draw	71
Participating EU member states	23

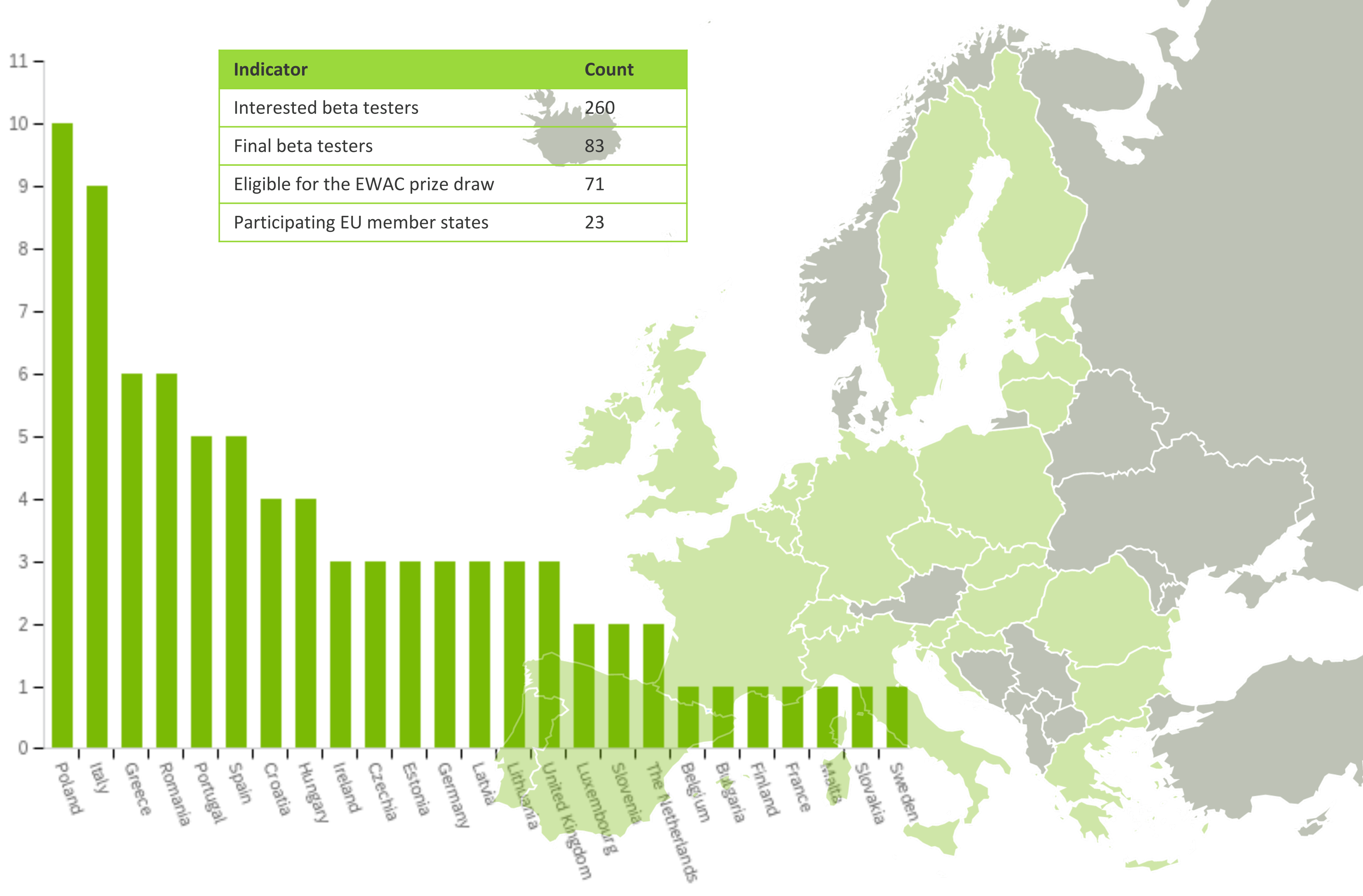

BETA TESTERS | Population demographics (II)

English native speaker

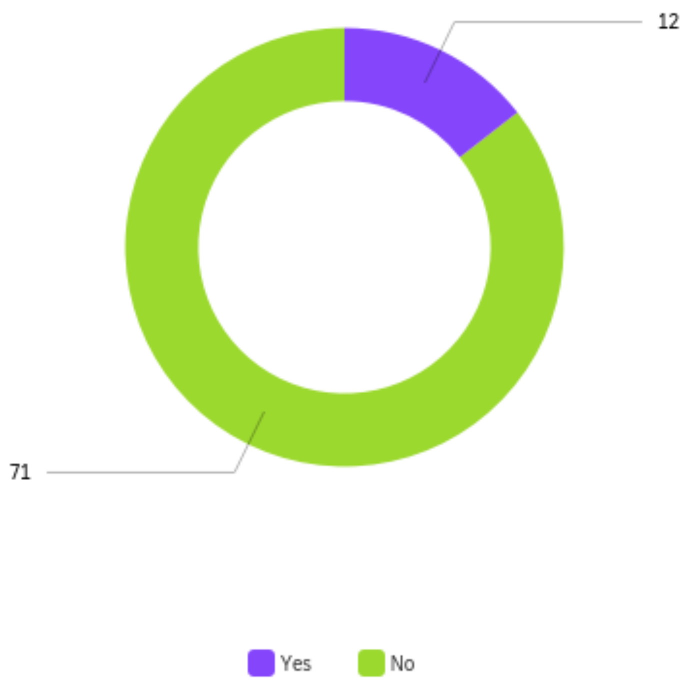

Answer	%	Count
Yes	14.46%	12
No	85.54%	71
**Total**	**100%**	**83**

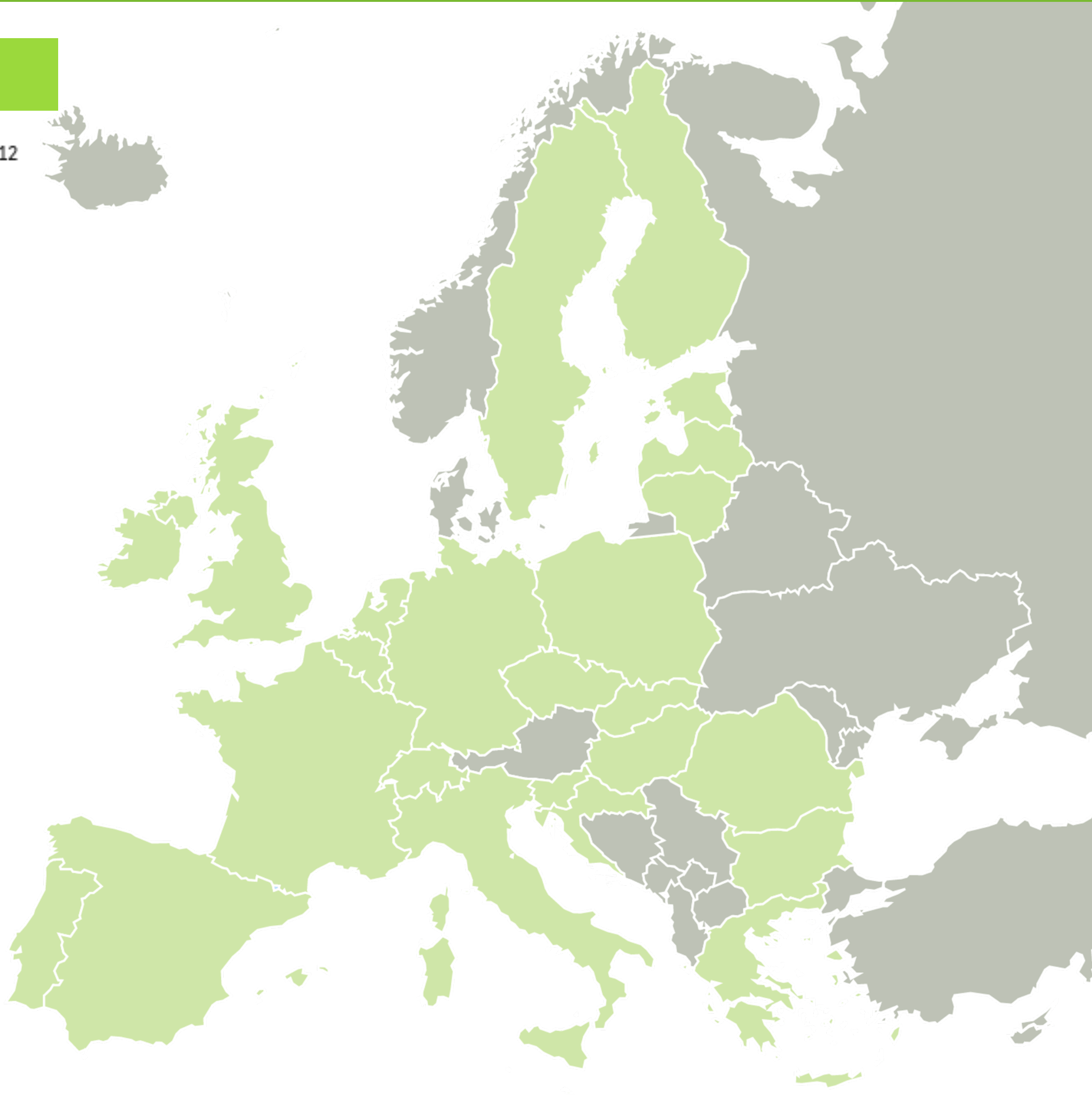

BETA TESTERS | Population demographics (III)

Age

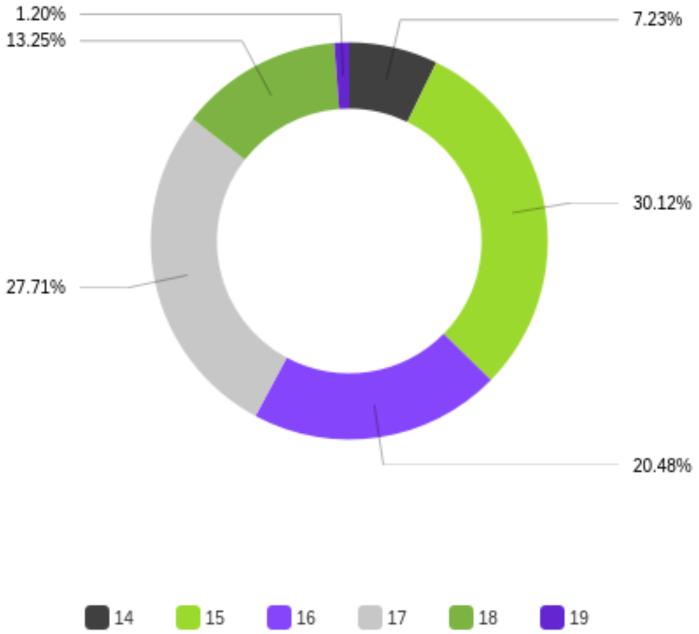

Answer	%	Count
14	7.23%	6
15	30.12%	25
16	20.48%	17
17	27.71%	23
18	13.25%	11
19	1.20%	1
**Total**	**100%**	**83**

Gender

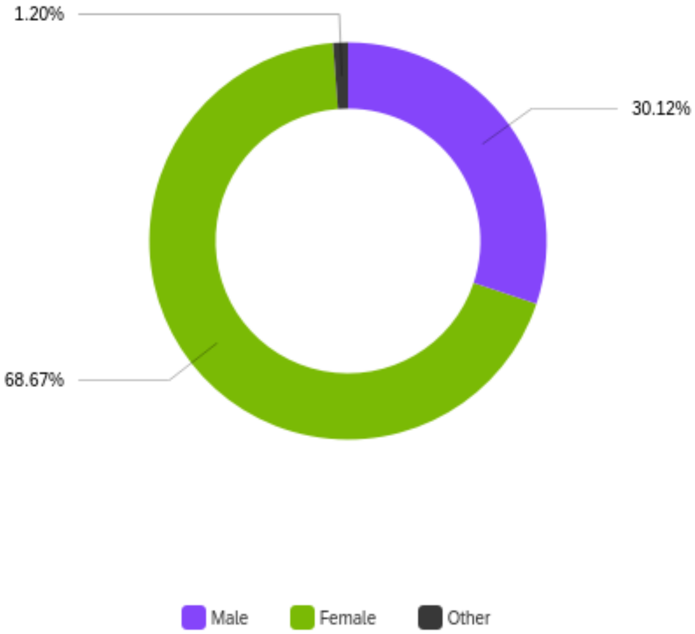

Answer	%	Count
Male	30.12%	25
Female	68.67%	57
Other	1.20%	1
**Total**	**100%**	**83**

5. Overall, how satisfied are you with the features you experienced in this early access of WASABY app?

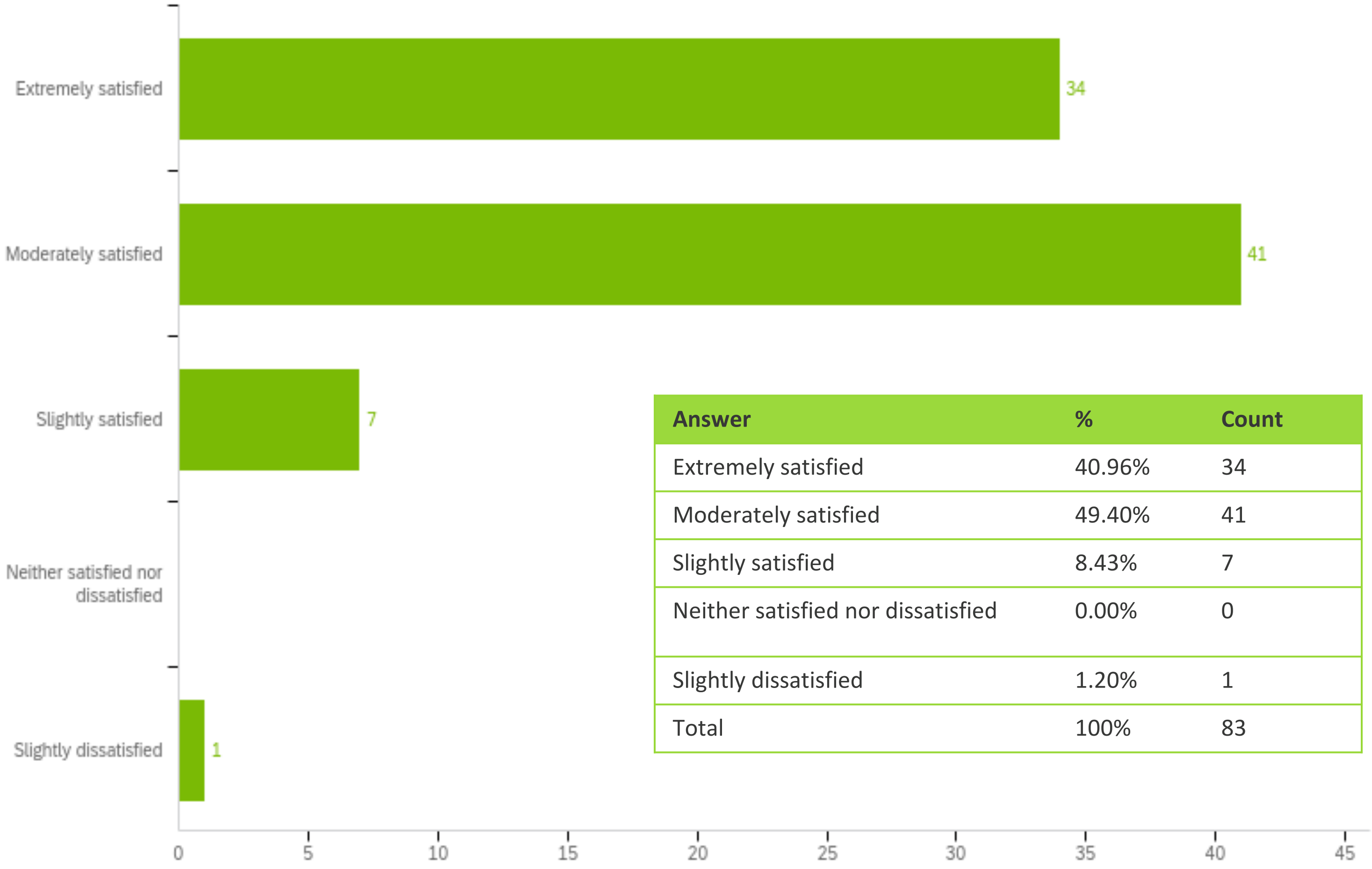

Answer	%	Count
Extremely satisfied	40.96%	34
Moderately satisfied	49.40%	41
Slightly satisfied	8.43%	7
Neither satisfied nor dissatisfied	0.00%	0
Slightly dissatisfied	1.20%	1
Total	100%	83

6. How easy or difficult was it to use WASABY app?

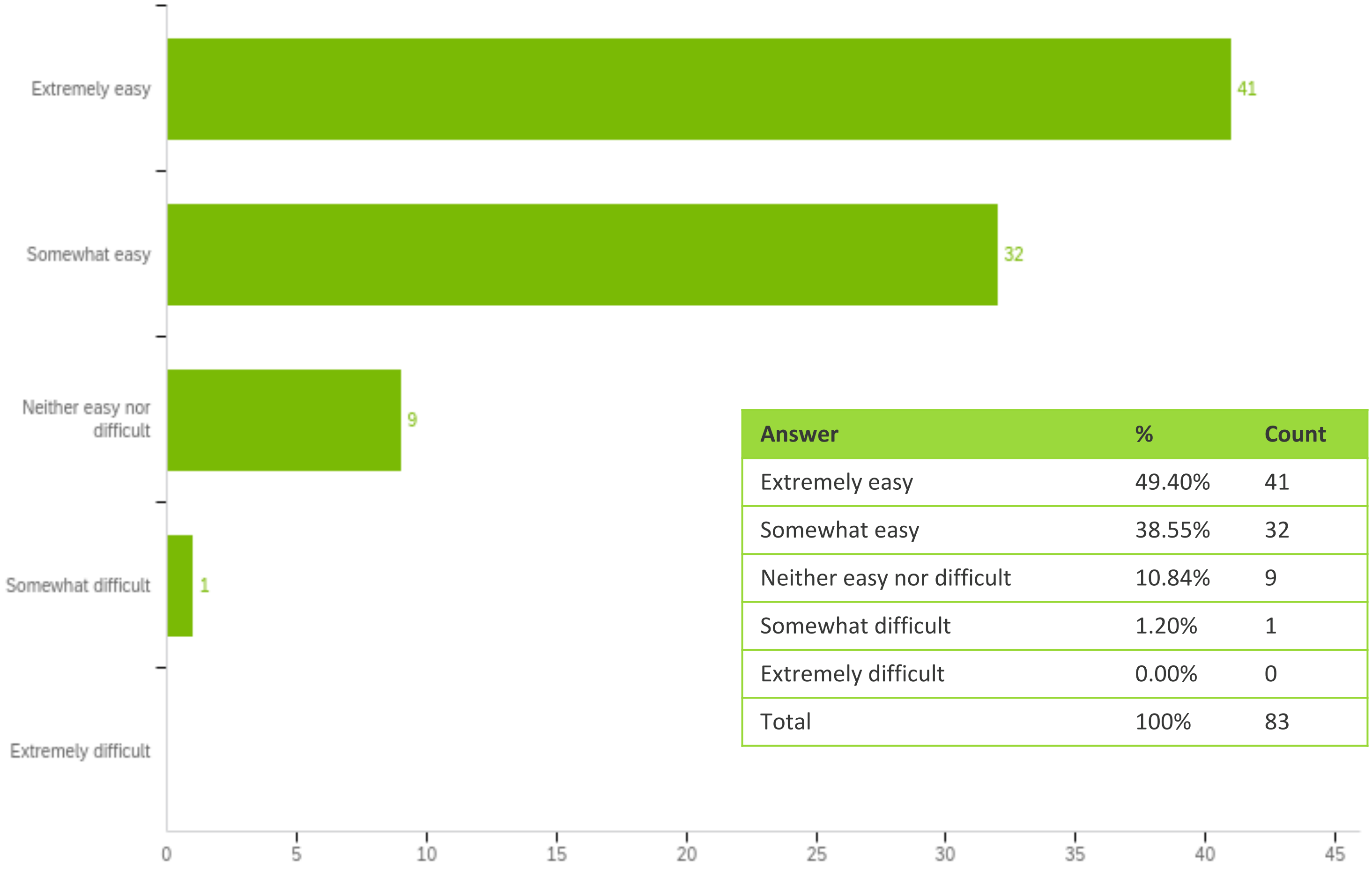

Answer	%	Count
Extremely easy	49.40%	41
Somewhat easy	38.55%	32
Neither easy nor difficult	10.84%	9
Somewhat difficult	1.20%	1
Extremely difficult	0.00%	0
Total	100%	83

6. How easy or difficult was it to use WASABY app? (by age and by native/ non-native English speakers)

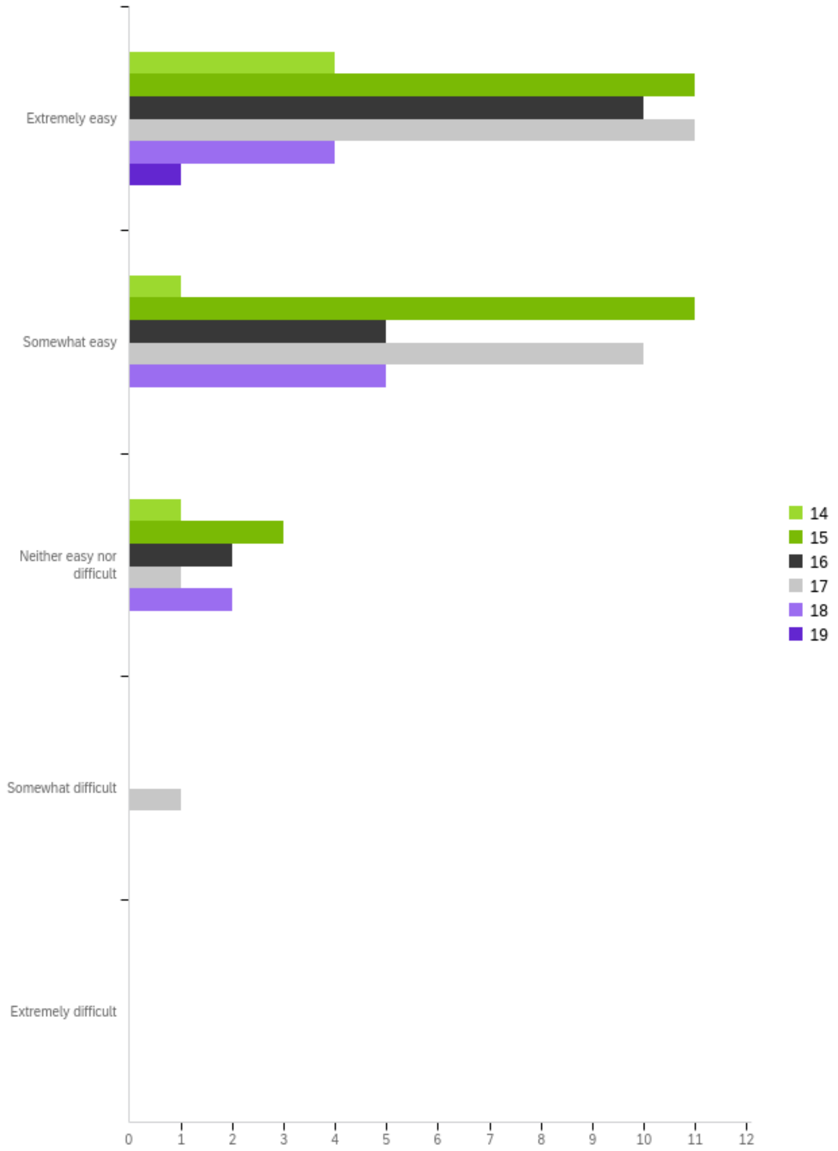

Answer	%	Count
Extremely easy	49.40%	41
Somewhat easy	38.55%	32
Neither easy nor difficult	10.84%	9
Somewhat difficult	1.20%	1
Extremely difficult	0.00%	0
Total	100%	83

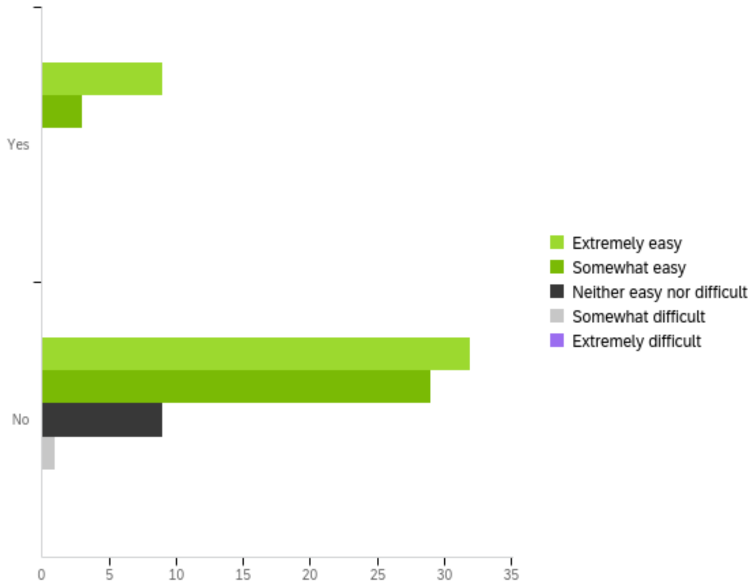

8. How much did you learn from WASABY app? (by age)

Answer	%	Count
A great deal	30.12%	25
A lot	46.99%	39
A moderate amount	19.28%	16
A little	3.61%	3
Nothing at all	0.00%	0
**Total**	**100%**	**83**

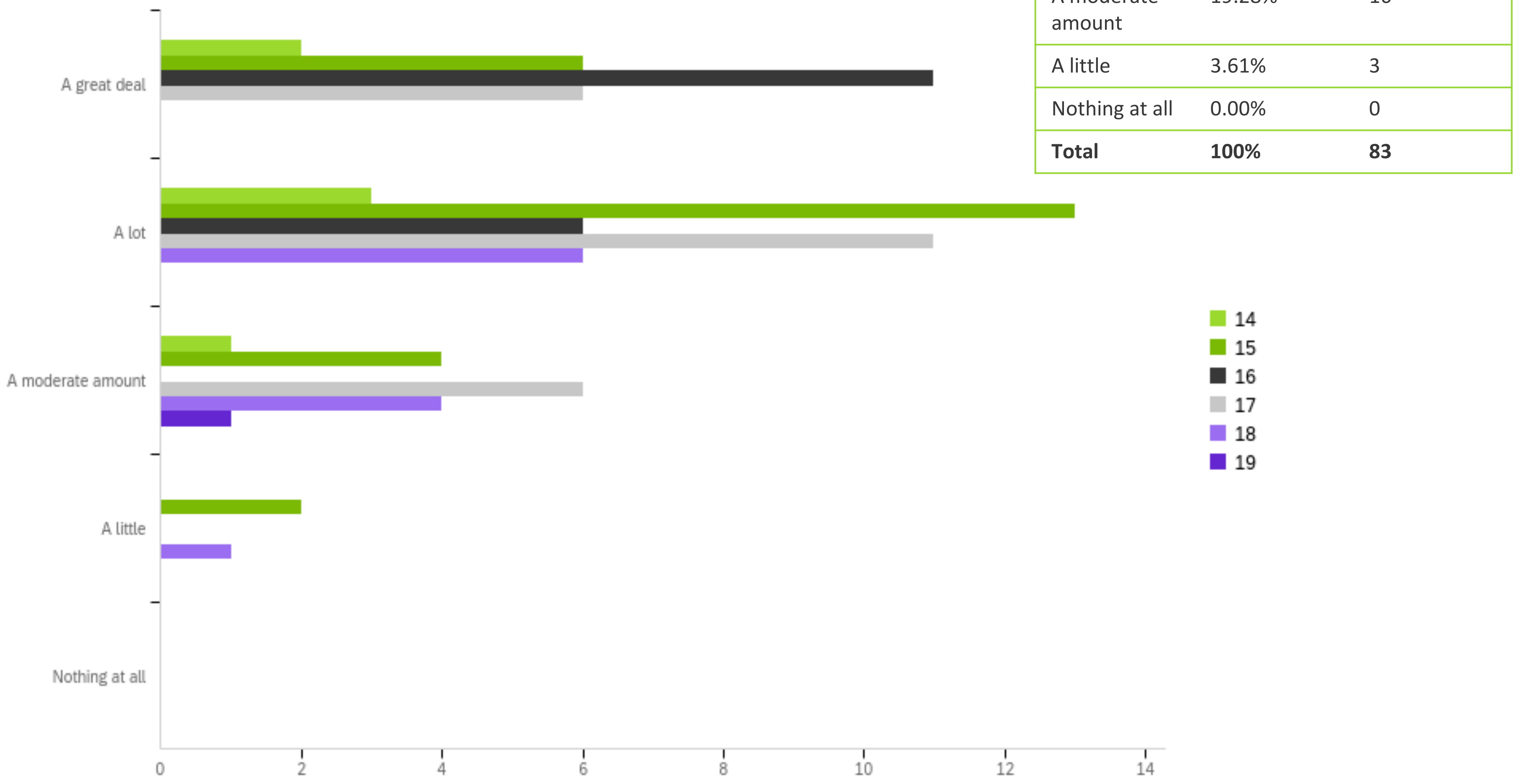

9. Please select a maximum of 3 modules that you found the most interesting and/or useful.

Top 3 modules

- M9: mythical causes of cancer
- M3: healthy eating
- M4: physical activity

Answer	%	Count
Module 1. Tobacco	9.54%	23
Module 2. Alcohol	8.30%	20
Module 3. Healthy eating	12.45%	30
Module 4. Physical activity	10.79%	26
Module 5. Healthy body weight	7.88%	19
Module 6. Vaccination, infection & cancer	9.96%	24
Module 7. Sun UV exposure	9.54%	23
Module 8. Breastfeeding	8.30%	20
Module 9. Mythical causes of cancer	14.52%	35
Module 10. Cancer prevention	8.71%	21
**Total**	**100%**	**241**

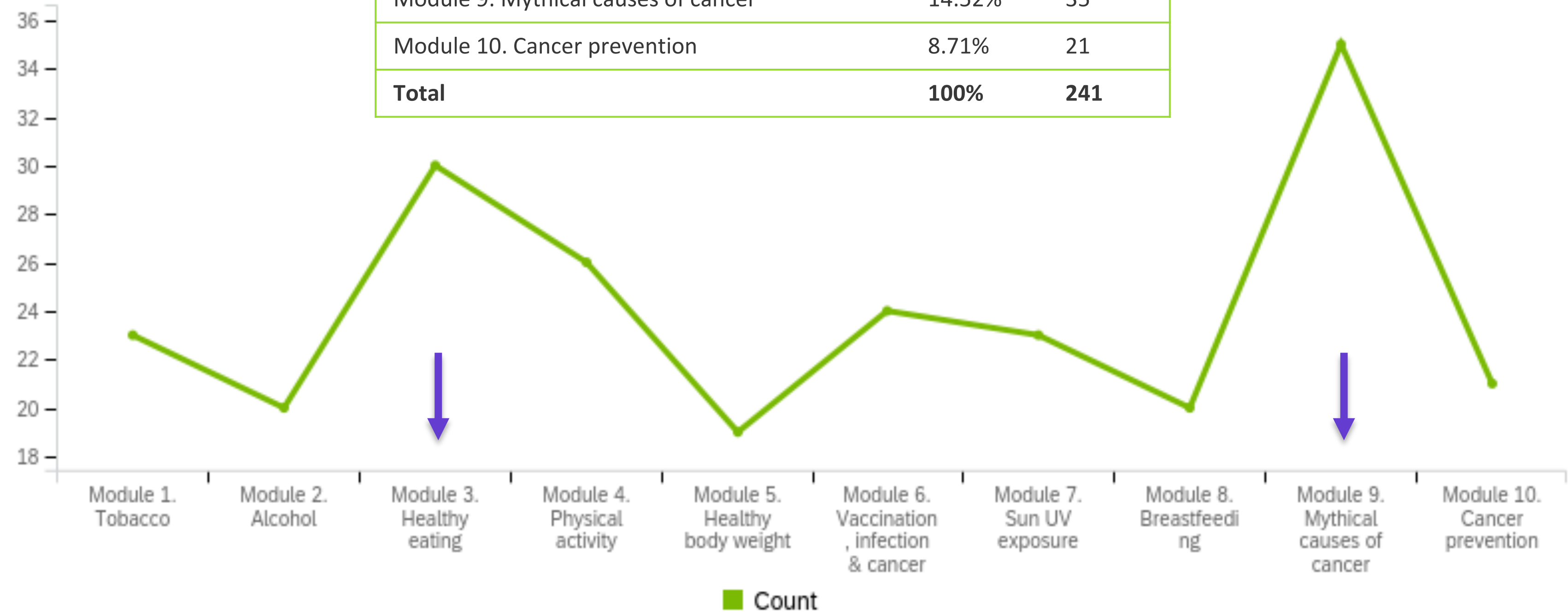

9. Please select a maximum of 3 modules that you found the most interesting and/or useful (by age & by gender)

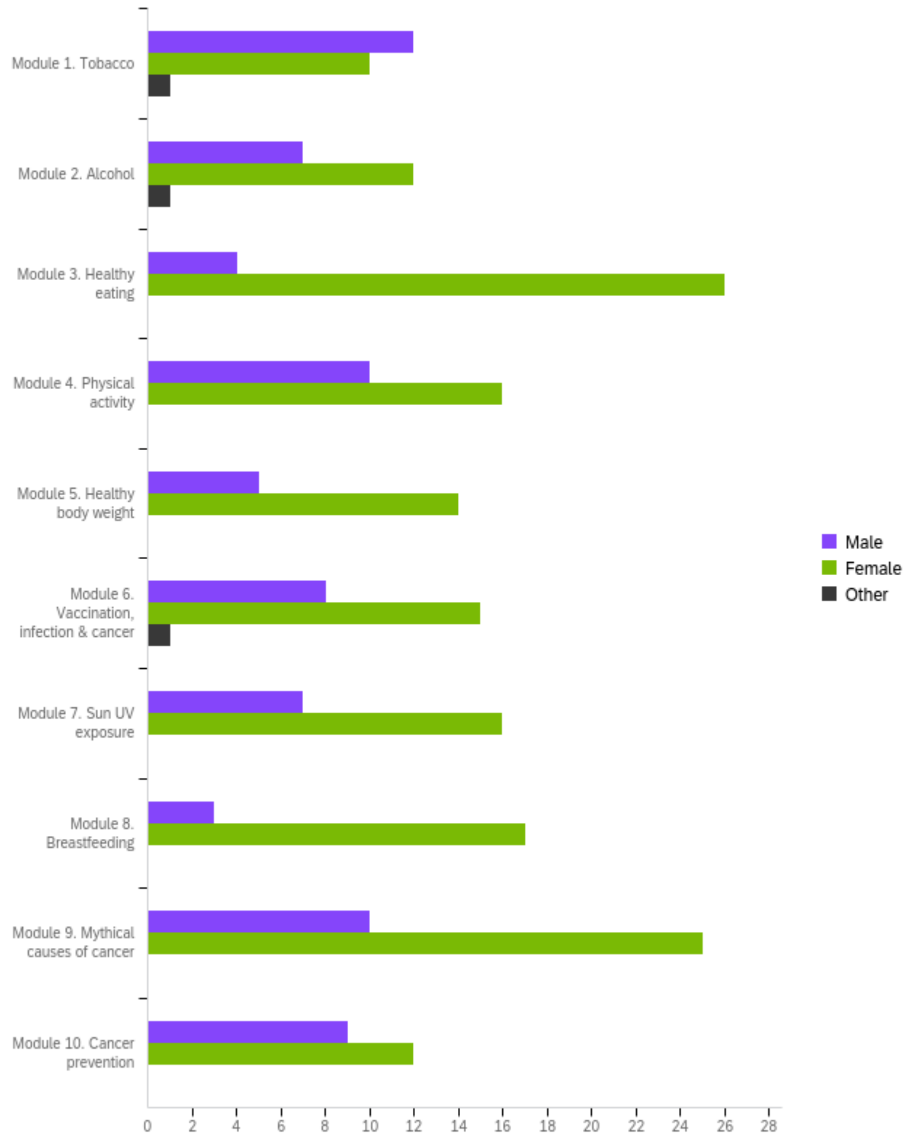

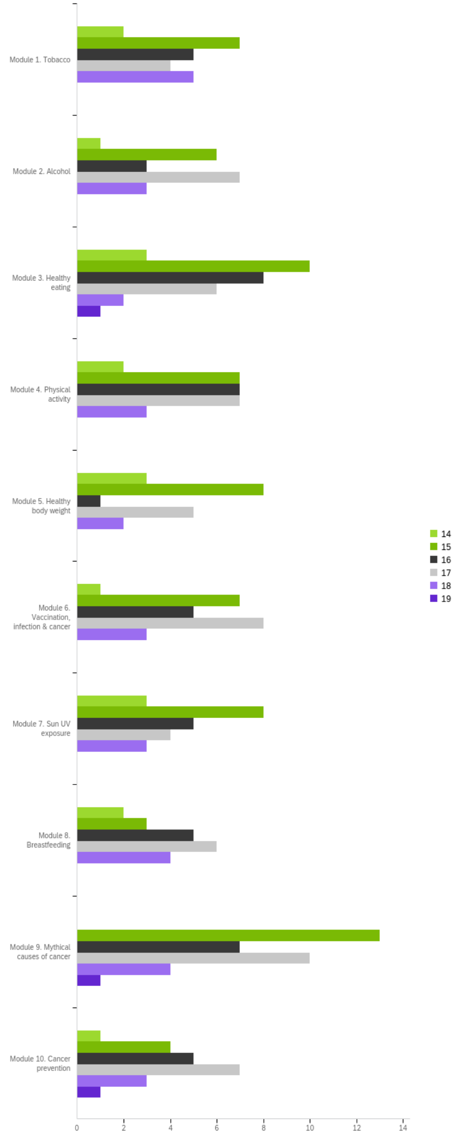

10. How challenging was the content showcased in WASABY app?

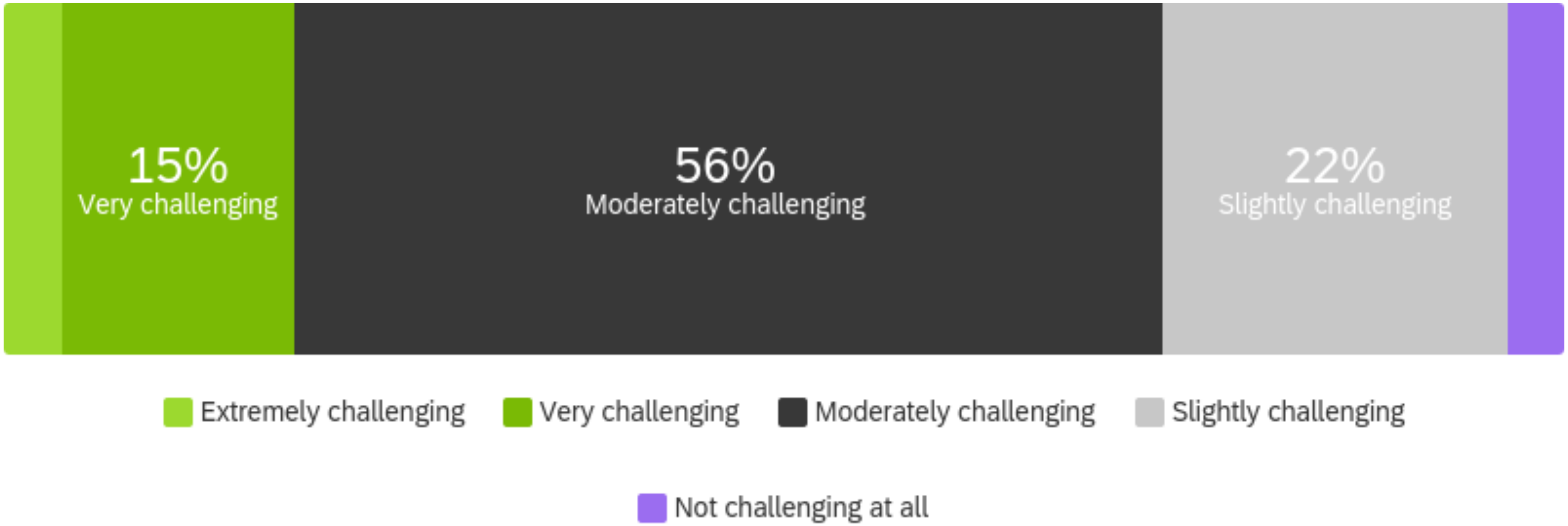

Minimum	Maximum	Mean	Std Deviation	Variance	Count
1.00	5.00	3.07	0.81	0.66	81

Answer	%	Count
Extremely challenging	3.70%	3
Very challenging	14.81%	12
Moderately challenging	55.56%	45
Slightly challenging	22.22%	18
Not challenging at all	3.70%	3
**Total**	**100%**	**81**

10. How challenging was the content showcased in WASABY app? (by age & gender)

14

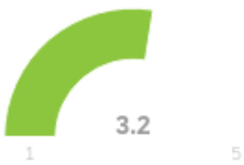

15

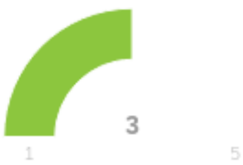

16

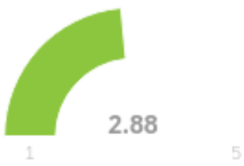

17

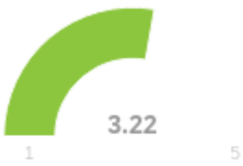

18

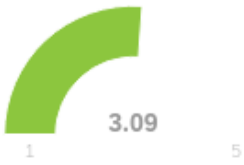

19

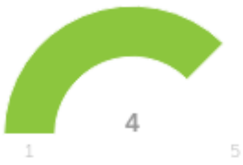

#	Answer	%	Count
1	Extremely challenging	3.70%	3
2	Very challenging	14.81%	12
3	Moderately challenging	55.56%	45
4	Slightly challenging	22.22%	18
5	Not challenging at all	3.70%	3
	**Total**	**100%**	**81**

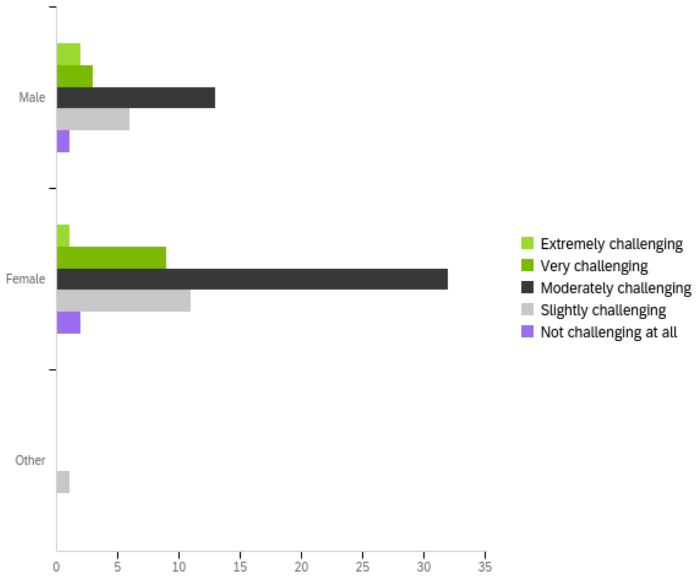

11. Please select a maximum of 3 modules that you found the most challenging.

Top 3 modules

- M6: vaccination, infection & cancer
- M10: cancer prevention
- M7: sun UV exposure

Answer	%	Count
Module 1. Tobacco	5.18%	10
Module 2. Alcohol	7.25%	14
Module 3. Healthy eating	6.22%	12
Module 4. Physical activity	7.77%	15
Module 5. Healthy body weight	10.36%	20
Module 6. Vaccination, infection & cancer	22.80%	44
Module 7. Sun UV exposure	11.40%	22
Module 8. Breastfeeding	7.77%	15
Module 9. Mythical causes of cancer	8.81%	17
Module 10. Cancer prevention	12.44%	24
**Total**	**100%**	**193**

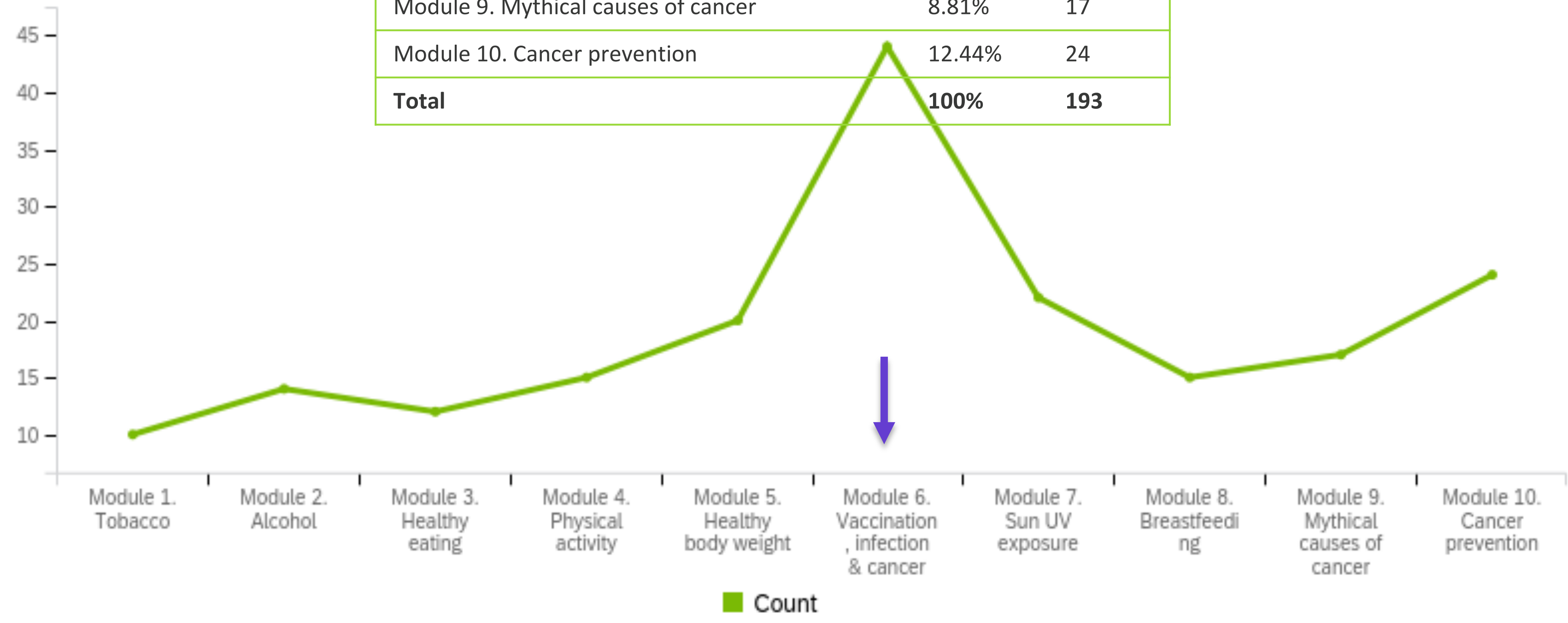

11. Please select a maximum of 3 modules that you found the most challenging (by age & gender)

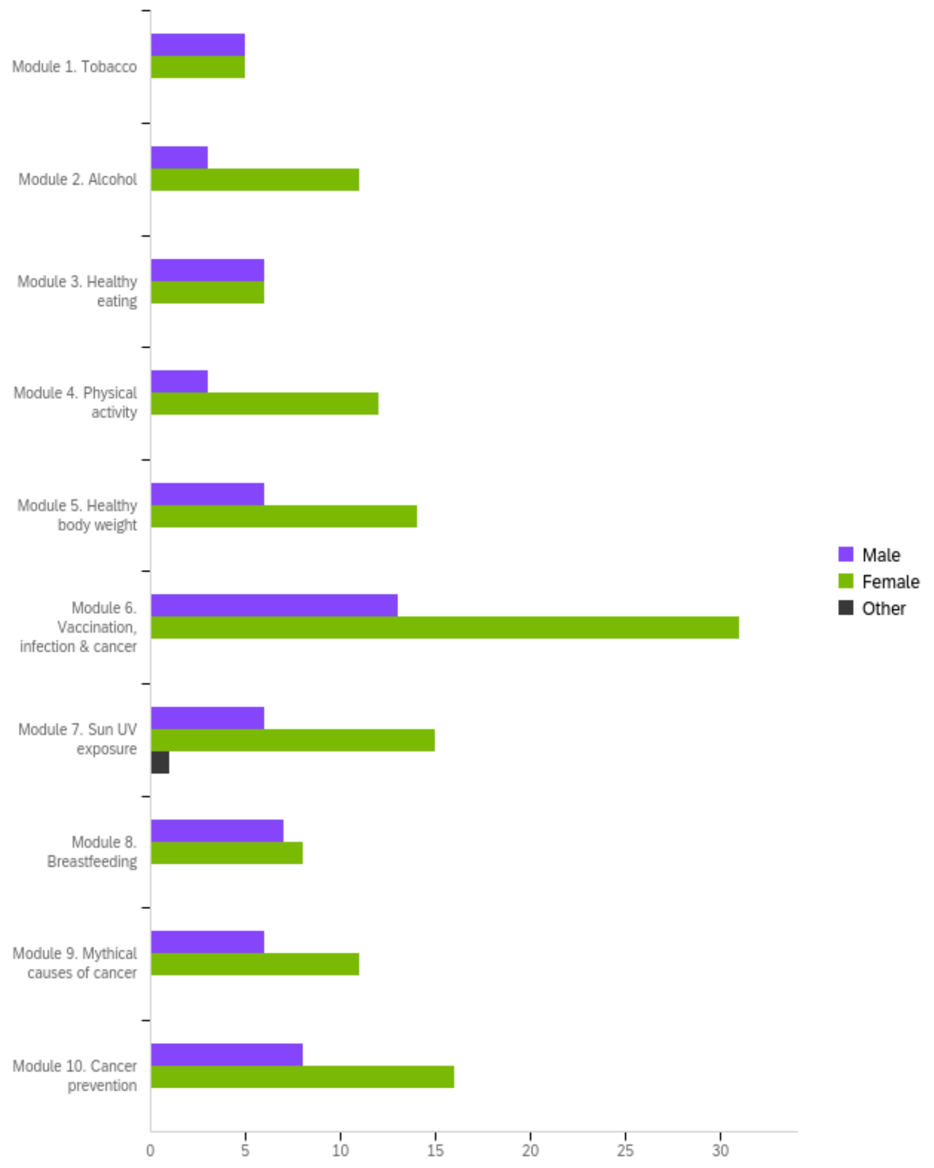

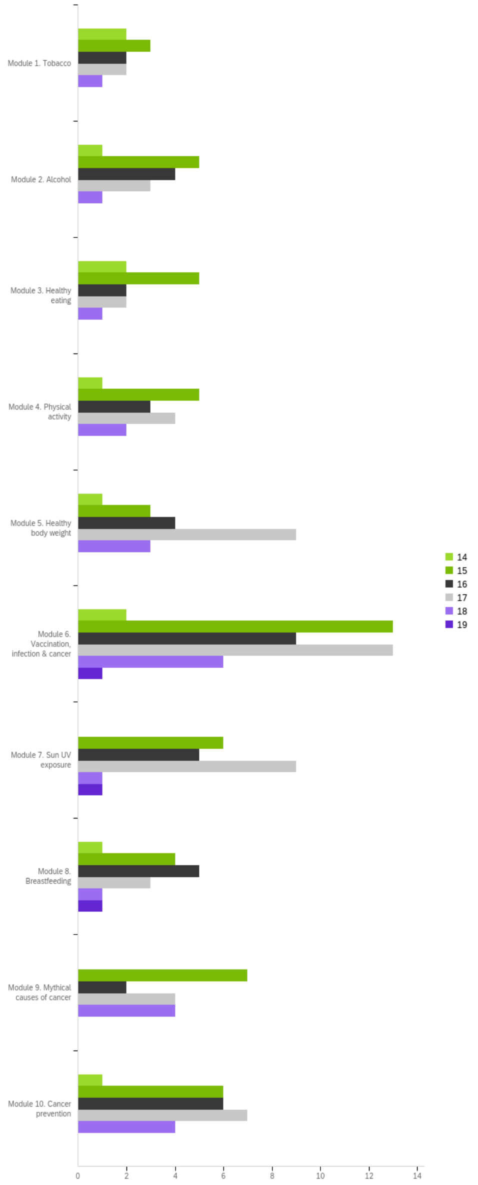

12. How well did WASABY app meet your expectations?

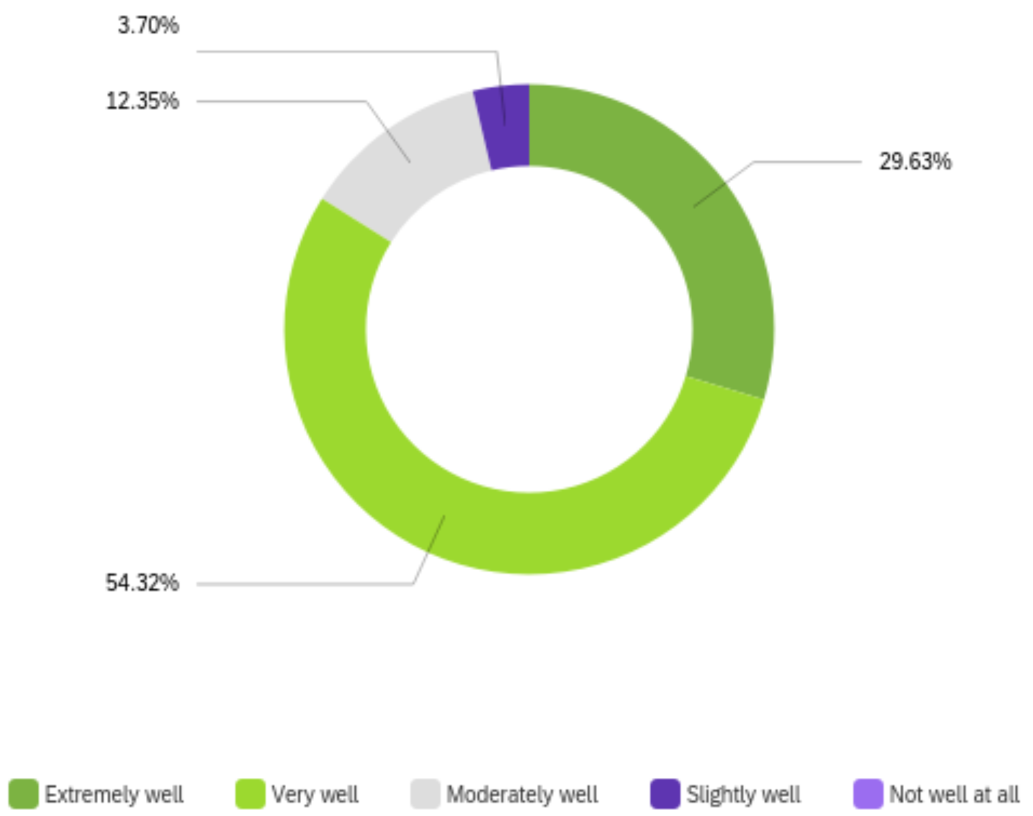

Answer	%	Count
Extremely well	29.63%	24
Very well	54.32%	44
Moderately well	12.35%	10
Slightly well	3.70%	3
Not well at all	0.00%	0
**Total**	**100%**	**81**

12. What did you like most about this WASABY app?

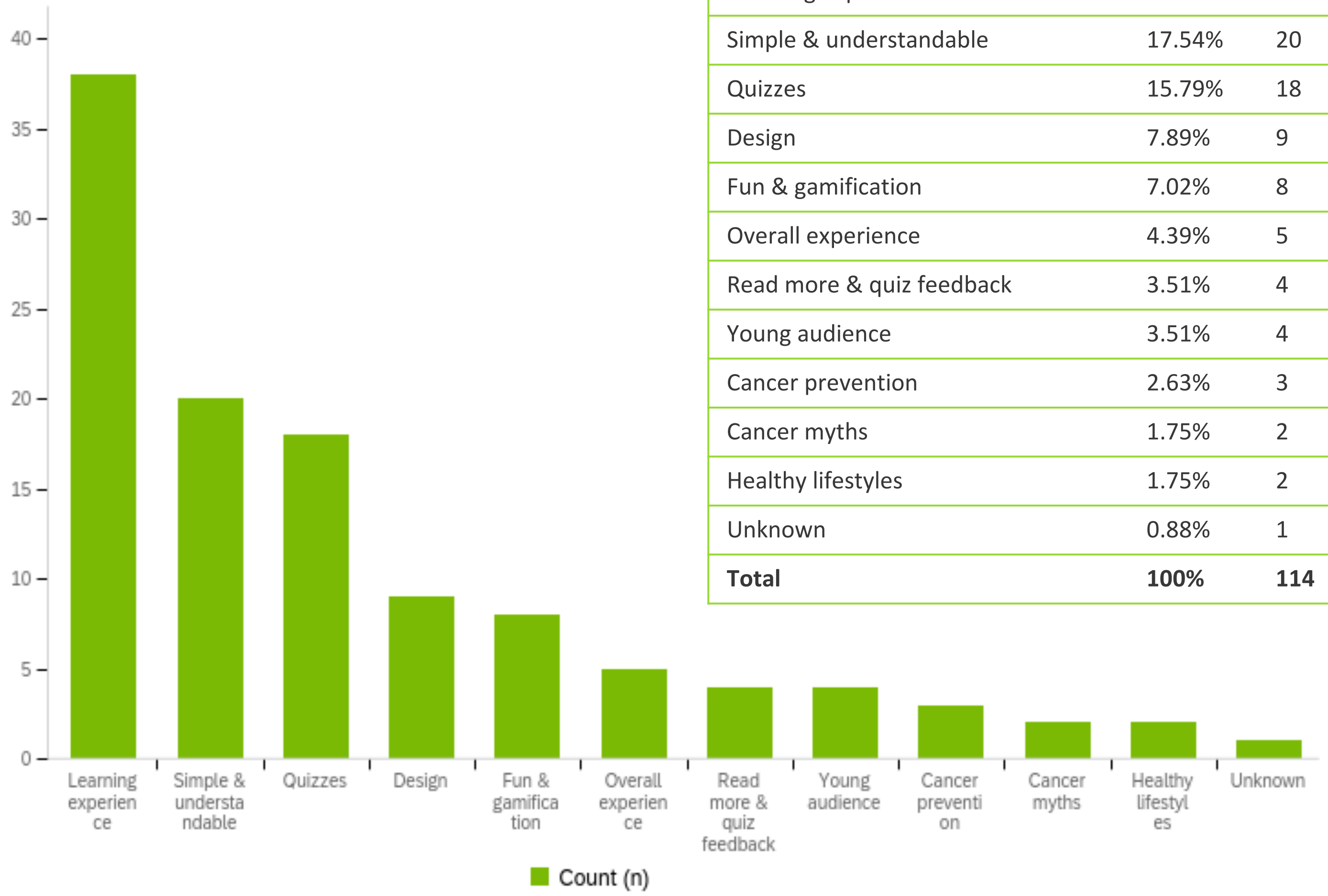

Answer	%	Count
Learning experience	33.33%	38
Simple & understandable	17.54%	20
Quizzes	15.79%	18
Design	7.89%	9
Fun & gamification	7.02%	8
Overall experience	4.39%	5
Read more & quiz feedback	3.51%	4
Young audience	3.51%	4
Cancer prevention	2.63%	3
Cancer myths	1.75%	2
Healthy lifestyles	1.75%	2
Unknown	0.88%	1
**Total**	**100%**	**114**

12. What did you like most about this WASABY app? (selected)

- I liked how it provided a lot of useful information to a normal person. Everyone is at a risk of cancer but there are factors that we can control to decrease the risk. I liked that the information was easy to read (easy language, good flow, logical order) and informative.
- Information is easy to understand, using is very simple.
- I liked the amount of information provided before each of the quizzes and the video that had a very pleasing animation quality that allowed for easier absorption of information for the quiz. Another strength of this app is that it is appropriate for a wide range of audience.
- That it covers everything about cancer and also debunks cancer-causing myths.
- I liked so much the clear information and the fact that if you was wrong it tells you why and give you the option to read more.

13. What did you like least about this WASABY app?

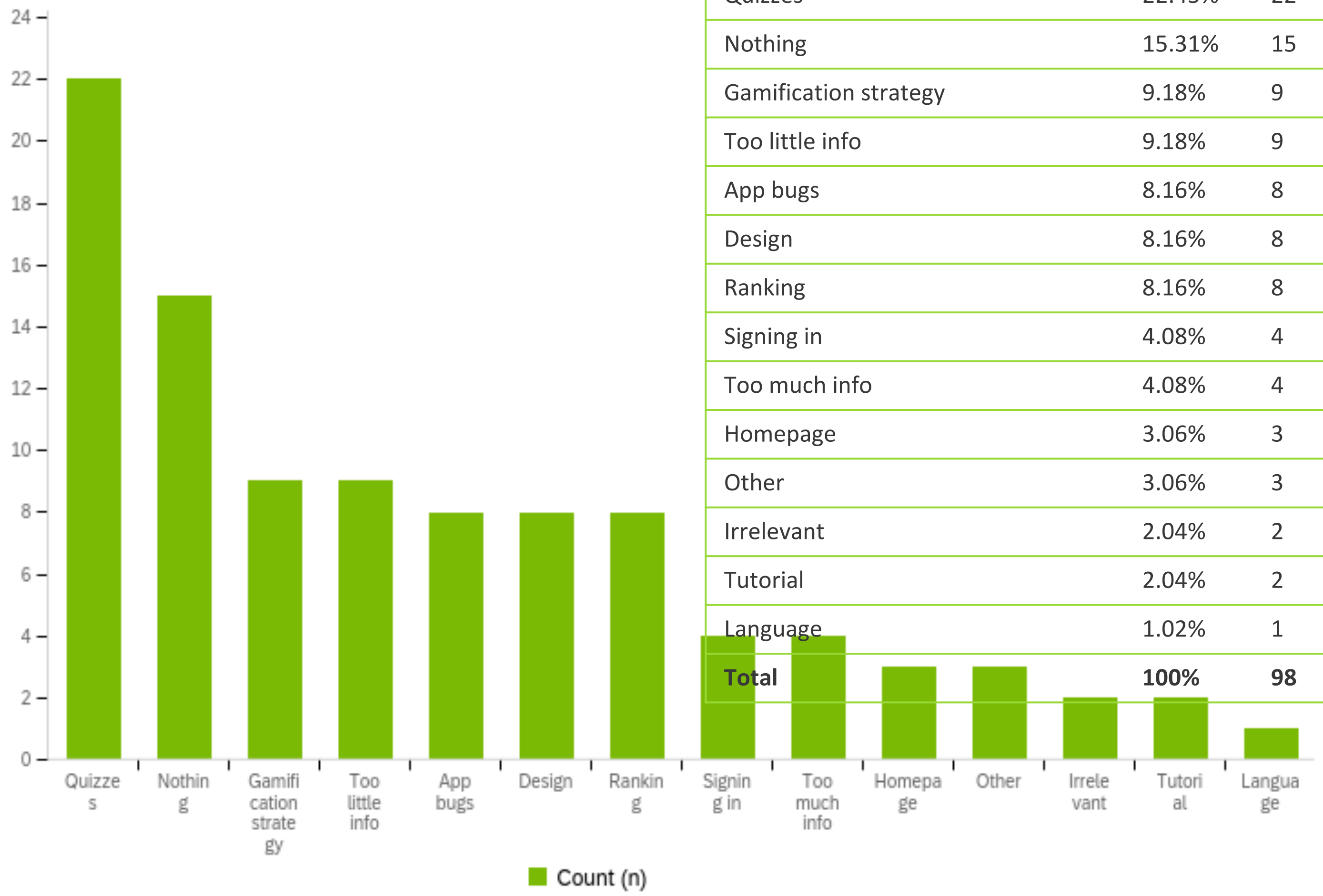

Answer	%	Count
Quizzes	22.45%	22
Nothing	15.31%	15
Gamification strategy	9.18%	9
Too little info	9.18%	9
App bugs	8.16%	8
Design	8.16%	8
Ranking	8.16%	8
Signing in	4.08%	4
Too much info	4.08%	4
Homepage	3.06%	3
Other	3.06%	3
Irrelevant	2.04%	2
Tutorial	2.04%	2
Language	1.02%	1
**Total**	**100%**	**98**

13. What did you like least about this WASABY app? (selected, I)

- I didn't really understand the ranking (…)
- Well maybe it wasn't that interactive like expected to be
- There were a few bugs in the app, for example I finished all the modules and the quiz but didn't get the 100% of questions answered award, it didn't put me on the leaderboard because my region says there's no students from there.
- Sometimes the text is really small on questions.
- I had a few problems with creating my account.
- My result did not appear on the ranking even though I managed to gather all the badges.
- (…) I sometimes couldn't see some options whole when I had to do the quiz, also sometimes the submit button would cover the last option.
- The "I have read it" button didn't work, so I couldn't read the information. I was only able to do the auto evaluation part (…)

13. What did you like least about this WASABY app? (selected, II)

- One thing I didn't like very much was that it didn't cover some topics in great detail.
- Too longer texts
- In the final test there were some questions I did not find an answer to in the progress section (e.g. how many calories does fat, alcohol, protein and carbohydrate have)
- That the quizzes weren’t as challenging as I would have thought.
- All options for 'All of the above' were the right answer, it makes it kinda boring
- Too many dry facts that one probably will not remember and that don't actually help me take action in preventing cancer in my day to day life.
- Not enough questions because they are interesting and test our knowledge
- The entire application was too short.

15. How do you think WASABY app could be improved? For example, are there topics that you would like to see covered, or do you have other suggestions for the team?

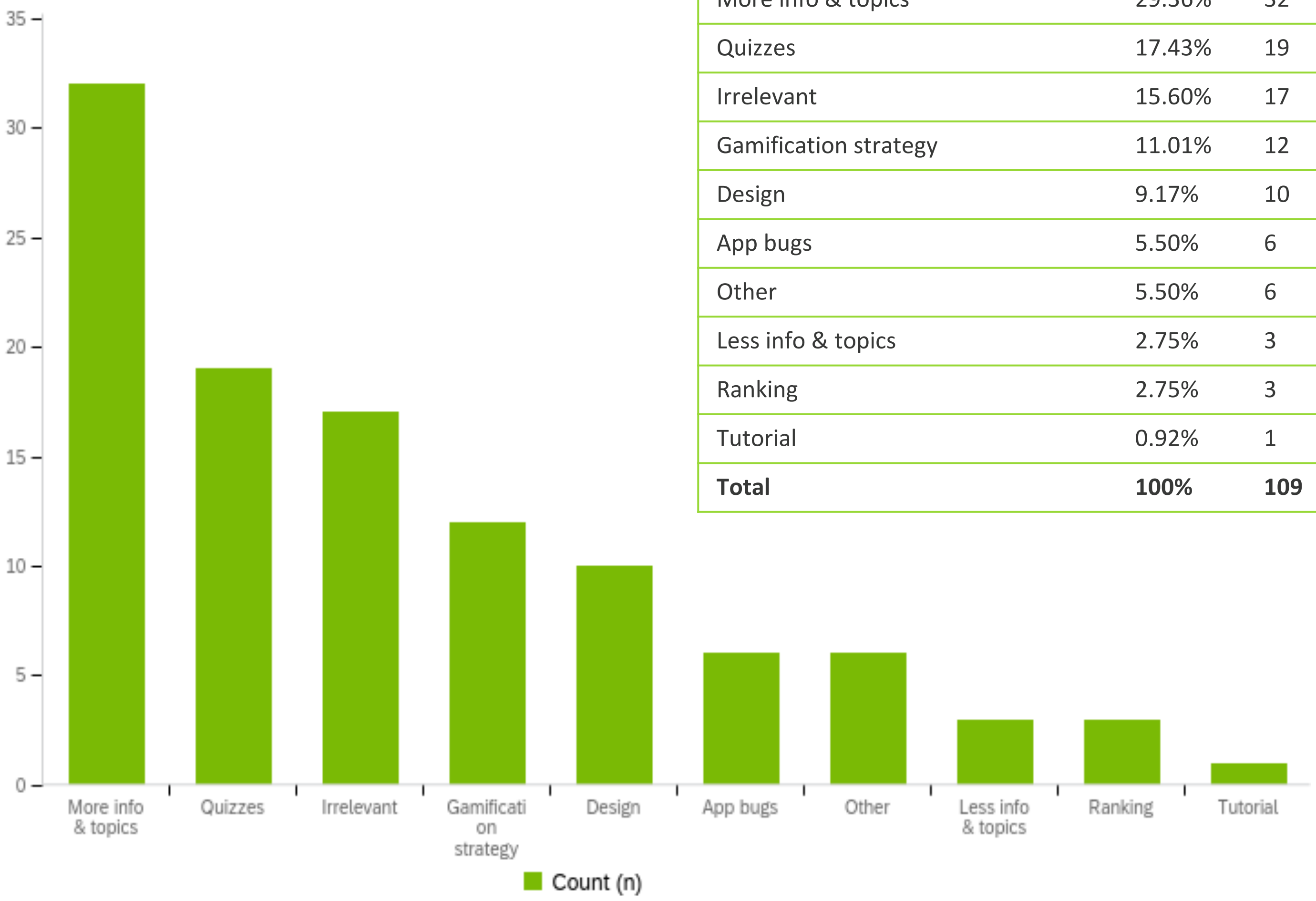

Answer	%	Count
More info & topics	29.36%	32
Quizzes	17.43%	19
Irrelevant	15.60%	17
Gamification strategy	11.01%	12
Design	9.17%	10
App bugs	5.50%	6
Other	5.50%	6
Less info & topics	2.75%	3
Ranking	2.75%	3
Tutorial	0.92%	1
**Total**	**100%**	**109**

15. How do you think WASABY app could be improved? For example, are there topics that you would like to see covered, or do you have other suggestions for the team? (selected, I)

- (…) it would be good if you could see the texts and do quizzes more than once.
- At the end of the quiz, they could put the percentage, and the points where it should improve, for people to get better and better.
- it would be easier to remember data if there were some graphs illustrating (…).
- Improve the visual, it isn't current enough
- After completing the final quiz with 100%, the achievement bar shows 75%. This is a fixable bug.
- Make the app more interactive. When I first logged into the app, I was surprised by how hard it was to find the actual questions. I thought I could just click the circles and go to the topics but that was not the case.
- (…) clearly organized as a learning app which may decrease student enthusiasm. The app should encourage people to use it and add more fast facts or small checkpoint questions along the article. Maybe some fun games would be beneficial since reading and getting quizzed can be a bit repetitive.

15. How do you think WASABY app could be improved? For example, are there topics that you would like to see covered, or do you have other suggestions for the team? (selected, II)

- Add questions that are more challenging and test the understanding of the topic rather than memory.
- I think it would be useful to put in a meal guide in the foods part and I would like to learn more about the mythical causes of cancer topic
- Your app could have a chat site, that is, for example, for people who smoke to talk to each other, or other people who need help and that talking makes them feel good with people like them. They can also make a website for news, for example or about alcohol or etc.
- Have topics differ for your gender, I would rather have the topic Breastfeeding switched with Prostate Cancer when I put down that I'm male.
- It would be great to use on a computer. Maybe have like user optional to change his background, when its dark and you open an app and its like a flashbang :-D
- You could add a daily reminder to check out the app

15. How do you think WASABY app could be improved? For example, are there topics that you would like to see covered, or do you have other suggestions for the team? (selected, III)

- (...) Another very important feature that could be added is whenever a selected answer is wrong the correct option should be highlighted, since sometimes it wasn't clear even from the articles provided. Additionally the app could provide a folder, in which one's incorrect answers could be stored and generate a separate quiz containing just those as a type of a review.
- Online use, team mode or more questions
- Maybe a challenges window would be great, something similar to ranking
- I think the information could be presented in a more engaging way - using more pictures, infographics and simpler language. For me it was alright, but I don't think a 14 year olds first priority will be to remember which types of HIV cause what. (...) Maybe there could be a separate section on tips on what you can do in your daily life to decrease cancer risk (...) it would be useful to have it all in a nice overview.
- Being able to click the icons on the main screen and then going to that page instead of clicking my progress

15. How do you think WASABY app could be improved? For example, are there topics that you would like to see covered, or do you have other suggestions for the team? (selected, IV)

- The guiding. It would be really helpful to have a guide at the beginning explaining everything and what is the use and the function of everything and how does it work.
- I would review the myths modul, there are two points I would like to be explained or elaborated on more-drinking from plastic bottles and using cleaning products. There are chemicals linked to cancer that are being used in producing plastic and that could be mentioned. I haven't read a study about drinking once from a plastic bottle but the topic of plastic and cancer could be improved. And the chemicals in products in general too-including cleaning AND cosmetics products. Not all cleaning products of course, but I think it's important to spread awareness about products and chemicals that we use for our skin too.
- There could be specific carcinogenic chemical compounds as topics.

16. Is there anything else you would like to tell us?

- I like how you made an app about cancer and everyone who is interested in it can use it.
- That's a great job, I find it very useful for teens.
- Thank you for deciding to make an app about cancer education for teens, I hope it'll get used in schools in the future.
- I find this initiative very interesting, helpful and positive for everyone to learn about health issues.
- It's fantastic app and I'll waiting for more topic and updates!

17. Do you feel WASABY app is ready for general release to all users?

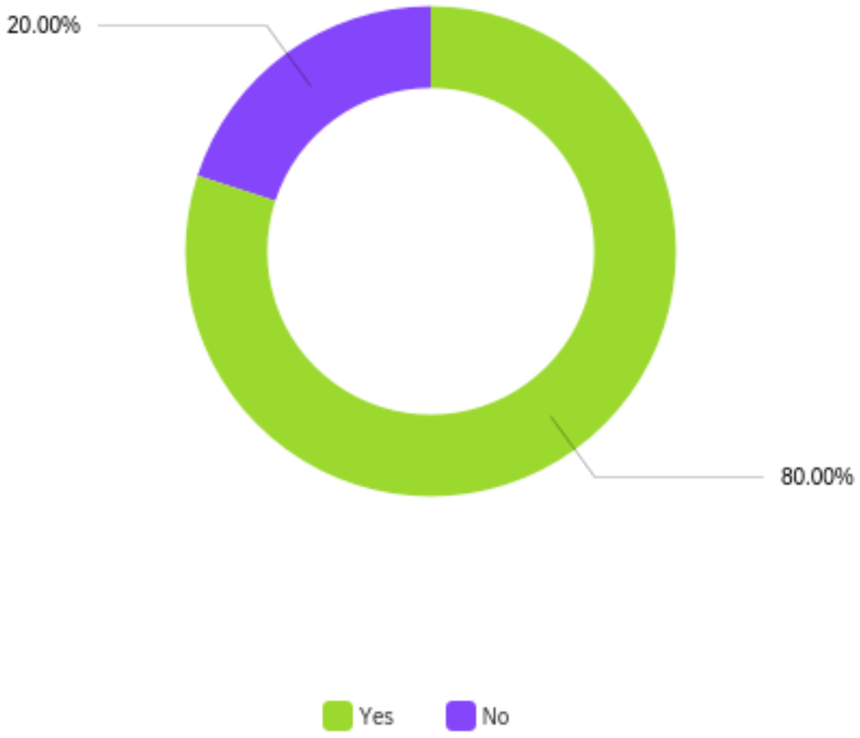

Answer	%	Count
Yes	80.00%	64
No	20.00%	16
**Total**	**100%**	**80**

18. Finally, how likely are you to recommend WASABY app to a friend or classmate?

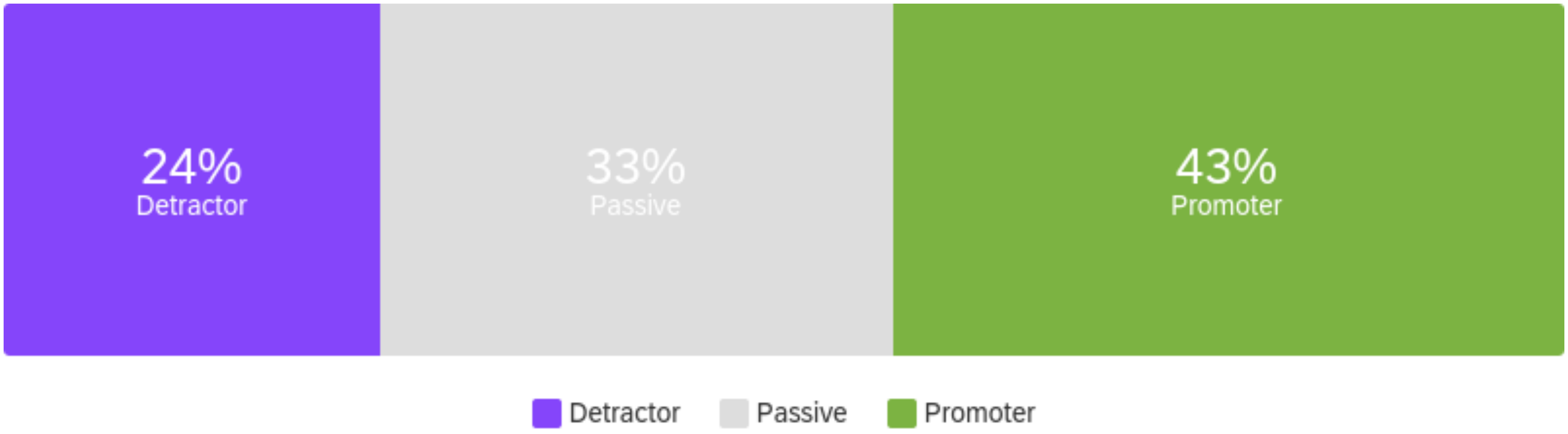

WASABY

Next steps

Items to discuss

- Feedback implementation
- iOS adaptation
- Alfa-testing (functionality)
- Project indicators
- Translation
- Timeline

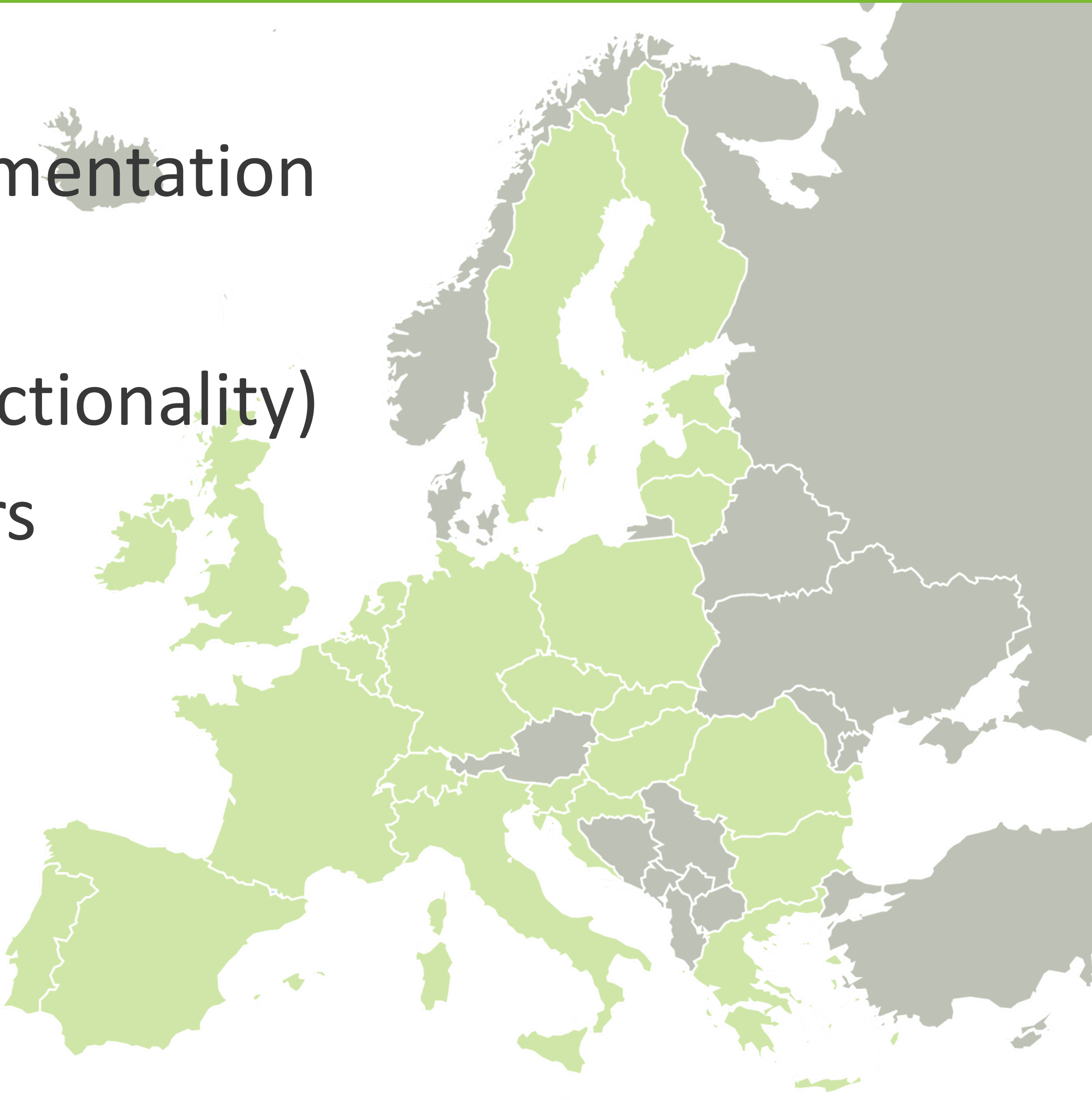

WASABY consortia set of indicators

Data from:

- Google analytics
- Flurry

**Specific Objective Number**	SO-5	
**Specific Objective**	Design courses on breast cancer risk factors awareness	
**Process Indicator(s)**	Target	**Value at M18 (May 2019)**
Number of target countries where promoting the online course	>=5	5 confirmed, 4 TBC
Number of participants per target country taking part in "alpha test" of online course	>=15	NA
Number of participants per target country taking part in final "beta test" of online course	>=15	NA
Mean age of participants to testing phase	14.5 yrs	NA
**Output Indicator(s)**	**Target**	**Value at M18**
Number of unique visits to online course per target country	>=5,000	NA
Engagement rate of Facebook page (or other social media metric)	>=50%	NA
Mean age of participants to online course	14.5 yrs	NA
**Outcome/Impact Indicator(s)**	**Target**	**Value at M18**
Number of unique visitors completing online course per target country	>=4,000	NA
Number of downloads of additional/complementary information by unique visitor after completion of course per country OR Number of unique visitors following hyperlinks to referral information / partner web pages after completion of course per country	>=1,000	NA

Indicative timeline

Item	Proposal date	Comments
Beta-testing (comprehension & adequacy)	27 May – 10 June 2020	Completed
Content adaptation	By July 12	
Translation	By the end of July	
Input of translated content into HTML files & quiz database	By early September	
Technical preps of translated content & beta testing feedback implementation	By early September	
Alpha-testing (functionality)	1 week over the second half of September	Prior to the alpha testing, all technical adaptations (including iOS) and translations should be done.
Alpha testing feedback implementation	By early October 2020	
Final WASABY app launch	By mid-late October 2020	
Final evaluation	October – December 2020	
Project finalisation	December 2020	Potentially extended over 6 months
Maintenance contract	To be discussed	

“Thank you for teaching me so much about cancer and how to prevent it. I will definitely be using this information in my life.”

Anonymous beta tester

Visit our website to learn more www.wasaby.it

Co-funded by
the Health Programme
of the European Union

EU funding disclaimer: This project has received funding from the 3rd European Union Health Programme 201-2020 under Grant Agreement PP-2-5-2016 (# 769767)
