# Supplementary material for: Development and Promotion of an mHealth App for Adolescents Based on the European Code Against Cancer: Retrospective Cohort Study
Source: JMIR Cancer. 2023 Nov 28;9:e48040. doi: 10.2196/48040 (PMC10716759; doi:10.2196/48040)
Supplement: Multimedia Appendix 2 [file cancer_v9i1e48040_app2.docx]

## Developing a Cancer Prevention Application for Adolescents: Results from a Pilot of a Mobile Health App Operationalizing the European Code Against Cancer

## 2.1 WASABY PRIVACY POLICY

1. **Introduction**

In accordance with the General Data Protection Regulation (EU) 2016/679 of the European Parliament and of the Council of April 27, 2016 ("GDPR"), and Law 3/2018 ‘Ley Orgánica de Protección de Datos y de Garantía de Derechos Digitales’ ("LOPDGDD", on *Protection of Personal Data and Digital Rights Guarantee)*, Salumedia Labs, S.L.U., (hereinafter, "Salumedia" or the data “Controller") offers you this Privacy Policy to inform you clearly and transparently about how we collect and process your personal data, through the Salumedia's Application (hereinafter the "Application").

1. **Identification of the Data Controller**

Salumedia Labs, S.L.U. (B91968685), with address of Calle Torcuato Luca de Tena 32, bloque 3, 5B, C.P. 41013, Seville (Spain), is the Data Controller for the processing of your personal data collected through the Application. You may contact us through our contact telephone number + 34 717 702 622 or through our email address: [legal@salumedia.com](about:blank)

1. **How we process your personal data**

The use of this application involves the processing of sensitive data related to the health of users. The treatment of these data is done with the purpose of offering an educational service on healthy lifestyles and cancer prevention. The data will also be used to carry out scientific research or for statistical purposes, always after the anonymisation of the data. Finally, and with the explicit consent of the user, the data may be used to carry out user profiling or big data, with the aim of offering a personalised learning strategy, adapted to the needs of each user. No automated decisions will be made that affect the personal data of each user.

| **When** | **Why** | **Legal Basis** | **What types of data?** |
| --- | --- | --- | --- |
| When you access and browse through the Application | Analyse, through Google Analytics and Furry, the use you make of the Application and your activity. | **Art. 6.1.a) GDPR:**  Express consent | IP address, geographic location, activity data, device data (fingerprint). |
| When you register as a User in the Application | To offer you a personalised service, developing a service adapted to your needs and preferences and perform a ranking with the best User scores from the Application associated with study centres. | **Art. 6.1.a) and b) GDPR:**   - Express consent - Execution of contractual provision | Technical data:  IP address, geographic location, activity data, Application usage data and the device (fingerprint).  Identification and contact information:  Username, date of birth, country, region, weight, height, physical activity, if you use tobacco or alcohol. |
| When you contact us by email, postcard, or phone | Respond to and resolve your queries, as well as maintain pre-contractual relationships. | **Art. 6.1.a) and b) GDPR:**   - Express consent - Execution of pre-contractual provisions | Name, surname(s), email address. |

1. **Duration of treatment. Retention of personal data**

We will keep the personal data you have provided for the time strictly necessary to fulfil each of the intended purposes. Specifically:

- **User account data:** During the time that your account as a WASABY user is active. When the contractual relationship ends, Salumedia will anonymise your personal data permanently, so it will be impossible to identify you as a User with the information stored by Salumedia. This will be performed unless there remains data that should still be retained because there is a specific legal retention obligation.
- **Data related to your navigation:** Salumedia will keep the data collected through the use of the Application in the aforementioned terms, for a period of two years.

1. **Communication and international transfer of personal data**

We do not share your personal data except with the suppliers with whom we work to be able to offer you the service, to comply with legal obligations, to respond to requests from judicial and/or administrative authorities, with the State Security Forces and Bodies and with companies, universities and foundations participating in the consortium formed by the Association of European Cancer Leagues (ECL) and its members, Fondazione IRCCS "Istituto Nazionale Tumori", Lubeck University, Caen University and Oncology Institute in Ljubljana.

No international transfers will be made to countries outside the European Economic Area (EEA), where the level of protection may not be equivalent to that granted by the Regulation, except with the United Kingdom and its transitional regime in force until December 31, 2020.

1. **Rights regarding the processing of personal data**

As a WASABY User you have the following rights in relation to the processing of your personal data:

- Right to request access to your personal data;
- Right to request rectification or suppression;
- Right to request the limitation of the processing of your personal data;
- Right to oppose the processing;
- Right to portability of the data in question, and
- The right to withdraw consent at any time.

Such rights can be exercised by sending an email to legal@salumedia.com, a written letter to the postal address of Salumedia, including as reference "Exercise of Rights". You can also make a complaint to the Spanish Agency for Data Protection on its website: [**www.aepd.es**](about:blank)**.**

**Latest version: 29 May 2020.**

**2.2. LEGAL NOTICE AND CONDITIONS OF USE**

**Information about the Owner of the Application**

In accordance with the provisions of article 10 of Law 34/2002, of July 11, de servicios de la sociedad de la información y de comercio electrónico (*on services of the information society and electronic commerce)*, the following information is provided:

| **Owner of the Application** | Salumedia Labs, S.L.U. (hereinafter Salumedia) |
| --- | --- |
| **Address** | **Calle Torcuato Luca de Tena 32, bloque 3, 5B, C.P. 41013, Seville (Spain).** |
| ***C.I.F.* [Company Tax ID]** | ESB91968685 |
| **Registration in the Commercial Registry of Seville** | Volume 5.442, folio 63, 1st inscription, SHEET SE-91-397 |
| **Contact details** | Telephone: + 34 717 702 622  info@salumedia.com  Avenida República Argentina 24, Edificio Torre de los Remedios, planta 5ª, módulo A. 41011 Seville, Spain |

**Terms and Conditions of Use of the Application**

**1. Purpose and scope of application**

These Terms and Conditions of Use regulate the access, navigation and use of Salumedia’s Application (hereinafter, the "Application"), as well as the responsibilities derived from the use of its Content ("Content" being understood to mean texts, graphics, drawings, designs, codes, software, photographs, music, videos, sounds, databases, images, expressions and information, as well as any other creation protected by national laws and international treaties on intellectual and industrial property). Likewise, "User" means the person accessing, browsing or using the services and activities, free or otherwise, developed in the Application.

Access or mere use of the Application by Users will be considered to imply their adherence to these Terms and Conditions published at any time. Consequently, the User is invited to read these Terms and Conditions carefully from time to time.

These Terms and Conditions of Use are the legal framework for the use of and access to the Application.

The use of this application involves the processing of sensitive data related to the health of users. The treatment of these data is done with the purpose of offering an educational service on healthy lifestyles and cancer prevention. The data will also be used to carry out scientific research or for statistical purposes, always after the anonymisation of the data. Finally, and with the explicit consent of the user, the data may be used to carry out user profiling or big data, with the aim of offering a personalised learning strategy, adapted to the needs of each user. No automated decisions will be made that affect the personal data of each user.

**2. Access**, **registration and use of the Application**

Access to the Application by Users is free of charge. To access the services offered through the Application, the User must create a personal profile by completing a registration form provided for this purpose. Upon completing this form, you will be entering into a binding contract with Salumedia.

All information that the User provides that contains pesonal data during said registration process will be subject to our **Privacy Policy** www.salumedia.com/wasaby-privacy. With regard to the information provided by the User, they will be responsible for such information being reliable, complete and truthful, and keeping it updated at all times.

The registered User will assume the responsibility for protecting their account, through a password that must be secure and secret. Therefore, the User will be responsible for the use made of both the User name and the password. In case of loss or theft of the access data to the account, the User must notify Salumedia immediately of this event, and change their password as soon as possible.

Access, navigation and use of the Application is the responsibility of the User, so they undertake to diligently observe any additional instructions given by Salumedia regarding the use of the Application and its contents, and not to use the Salumedia service, nor its Content in any way that is not expressly authorised by these Conditions.

Users must be at least 14 years old to use the Application. Therefore, the use of the Application by users under 14 years of age is not allowed.

The User acknowledges that neither Salumedia nor the Association of European Cancer Leagues is a medical organization and that the Application does not provide medical advice, diagnosis, emergency care or prescription of drug treatments. The User assumes that the purpose of the Application is the provision of information on healthy lifestyles and cancer prevention. The User is warned that the use of the Application cannot under any circumstances replace the face-to-face consultation of a suitably qualified medical professional.

The User accepts the content of these Conditions, so they accept that failure to observe them may cause the immediate termination of their relationship with Salumedia and, consequently, the deletion of their account.

In particular, the User will refrain from:

- Using the Content included in the Application for purposes or effects contrary to the law, morality and generally accepted good customs or public order;
- Reproducing or copying, distributing, allowing public access through any form of public communication, transforming or modifying the Content, unless with authorisation from the appropriate copyright holder or when legally allowed;
- Using the Content and, in particular, the information obtained through the Application for advertising purposes;
- Providing any personal information or in relation to the use and access to the Application that is false or has been illegally obtained.

**3. Rights over the Application and its Content**

The Content and graphic elements of the Application are owned by ECL (Association of European Cancer Leagues). Notwithstanding the above, Salumedia is the owner of the intellectual property rights over the Application and the other elements it comprises. In no case will it be understood that the mere access or navigation of the User in the Application implies any type of transmission, license or total or partial transfer of said rights by Salumedia in favour of the User, nor that the User is authorised to use or exploit such rights publicly.

The deletion or manipulation of the copyright notice ("copyright") and any other identification or recognition of exclusive ownership of Salumedia's rights in relation to the Application or the protected Content found in it is totally forbidden.

Likewise, it is strictly forbidden to reproduce, distribute, publicly communicate, reuse, extract, send by mail, modify or in any way publicly use all or part of the Content included in the Application, without the express consent of Salumedia.

In the event that, on the occasion of access or use of the Application, the User sends or transmits to Salumedia some type of content protected by intellectual or industrial property rights, the User undertakes to previously acquire all and any authorisations, licenses or rights of use that are necessary to guarantee that Salumedia may have untroubled use of said content.

**4. Responsibilities and Warranty**

Salumedia manages the Application diligently and, therefore, seeks at all times to maintain complete and accurate information of all the services it offers, updating it at all times. However, it is possible that, occasionally, there may be some disagreement between the information offered in the Application and the reality of the service actually provided.

Salumedia has always taken appropriate measures, in accordance with the up-to-date state of the art, to ensure the proper operation of the Application and prevent the existence of viruses or computer programs that may cause harm to Users.

However, computer security is not infallible and is exposed to certain risks as a result of the inevitable activity carried out by hackers and programmers of viruses or harmful programs. Therefore, although Salumedia makes every effort to avoid these types of programs, and constantly updates its computer and systems security, Salumedia or ECL cannot guarantee, nor is it responsible for, the damages suffered by the User as a consequence from:

- Failures or interruptions of the Application service;
- Viruses or computer programs or any elements designed for cyber-attack or infiltration into User computer systems that, inadvertently, may have been installed, owing to or as a consequence of the access to the Application by the User;
- Eventual vulnerability of the Application and the security measures adopted therein;

**5. Links to other websites**

In the Application, the User can find links to other Applications or Websites managed by third parties. Salumedia does not have the power to control all the information or content provided by third parties, so it cannot assume any responsibility for their quality and reliability, or the veracity or adequacy of the content offered.

The establishment of any type of link by the Application to another Website or third-party Application does not imply that there is any kind of relationship, collaboration or dependence between Salumedia and the owner of said Website or Application.

**6. Duration and Modification**

Salumedia, upon previous consultation with ECL, may modify these Terms and Conditions, in whole or in part, by publishing any change in the same way as these general conditions.

Likewise, Salumedia will do everything possible to ensure the continuity of the service and the operation of the Application. However, and to the extent permitted by law, Salumedia may, upon previous consultation with ECL terminate, suspend or interrupt, at any time and without prior notice, access to the Content of the Application, with no possibility for the User to seek any compensation, either for technical or maintenance difficulties.

**7. Declaration of Invalidity**

The eventual declaration of invalidity of any one of the provisions outlined in these Terms and Conditions will not affect other provisions that are not affected by such declaration.

**8. Applicable Law Competent Jurisdiction**

These Terms and Conditions are governed by Spanish Law, notwithstanding the application of any other regulations issued by a competent authority.

Any disagreement between the User and Salumedia about the validity and applicability of any provision contained in these Terms and Conditions will be resolved judicially, in accordance with the competition rules established in the applicable regulations.

**Latest version: 29 May 2020**
