# Supplementary material for: Development and Promotion of an mHealth App for Adolescents Based on the European Code Against Cancer: Retrospective Cohort Study
Source: JMIR Cancer. 2023 Nov 28;9:e48040. doi: 10.2196/48040 (PMC10716759; doi:10.2196/48040)
Supplement: Multimedia Appendix 3 [file cancer_v9i1e48040_app3.docx]

WASABY App store Key Performance Indicators (KPIs) for the period of 4 February to 30 June, 2021, during which it received dedicated promotion through social media channels.

| **Key Performance Indicators** | **Android** | **iOS** | **Overall** |
| --- | --- | --- | --- |
| 1. Product page views | 834 | 2592 | 3426 |
| 2. Product installations | 594 | 515 | 1109 |
| 3. Conversion rate | 73.02% | 19.87% | 32.37% |
| 4. Deletions | 466 | 179 | 645 |
| 5. Crashes | 82 | 180 | 262 |
| 6. Sessions per active device | - | 3.32 | 3.32 |
| 7. Average rating | 3.9 | 5 | 3.97 |
